# Structural and functional insights into the *Pseudomonas aeruginosa* glycosyltransferase WaaG and the implications for lipopolysaccharide biosynthesis

**DOI:** 10.1016/j.jbc.2023.105256

**Published:** 2023-09-15

**Authors:** Emma R. Scaletti, Pontus Pettersson, Joan Patrick, Patrick J. Shilling, Robert Gustafsson Westergren, Daniel O. Daley, Lena Mäler, Göran Widmalm, Pål Stenmark

**Affiliations:** 1 Department of Biochemistry and Biophysics, Stockholm University, Stockholm, Sweden; 2 Department of Organic Chemistry, Arrhenius Laboratory, Stockholm University, Stockholm, Sweden

**Keywords:** *Pseudomonas aeruginosa*, WaaG, glycosyltransferase, lipopolysaccharide, X-ray crystallography, NMR

## Abstract

The glycosyltransferase WaaG in *Pseudomonas aeruginosa* (PaWaaG) is involved in the synthesis of the core region of lipopolysaccharides. It is a promising target for developing adjuvants that could help in the uptake of antibiotics. Herein, we have determined structures of PaWaaG in complex with the nucleotide-sugars UDP-glucose, UDP-galactose, and UDP-GalNAc. Structural comparison with the homolog from *Escherichia coli* (EcWaaG) revealed five key differences in the sugar-binding pocket. Solution-state NMR analysis showed that WT PaWaaG specifically hydrolyzes UDP-GalNAc and unlike EcWaaG, does not hydrolyze UDP-glucose. Furthermore, we found that a PaWaaG mutant (Y97F/T208R/N282A/T283A/T285I) designed to resemble the EcWaaG sugar binding site, only hydrolyzed UDP-glucose, underscoring the importance of the identified amino acids in substrate specificity. However, neither WT PaWaaG nor the PaWaaG mutant capable of hydrolyzing UDP-glucose was able to complement an *E. coli* Δ*waaG* strain, indicating that more remains to be uncovered about the function of PaWaaG *in vivo*. This structural and biochemical information will guide future structure-based drug design efforts targeting PaWaaG.

A common and defining feature of Gram-negative bacteria is the architecture of the cell envelope that contains two membranes, a cytoplasmic inner membrane and an outer membrane (OM), separated by the periplasmic space and the peptidoglycan layer. The OM is asymmetric, and the outer leaflet contains lipopolysaccharides (LPS) whereas the inner leaflet contains phospholipids. LPS anchored in the membrane acts as a protective layer (1) and as a virulence factor (2). It consists of three parts: (i) lipid A comprising a disaccharide acylated with up to eight fatty acids of different complexity (3), (ii) a core region containing about a dozen sugar residues and (iii) an O-antigen polysaccharide usually made up of 10 to 25 repeating units having 2 to 7 sugar moieties depending on biosynthetic pathway and serogroup (4, 5) (Fig. 1*A*).

**Figure 1:**
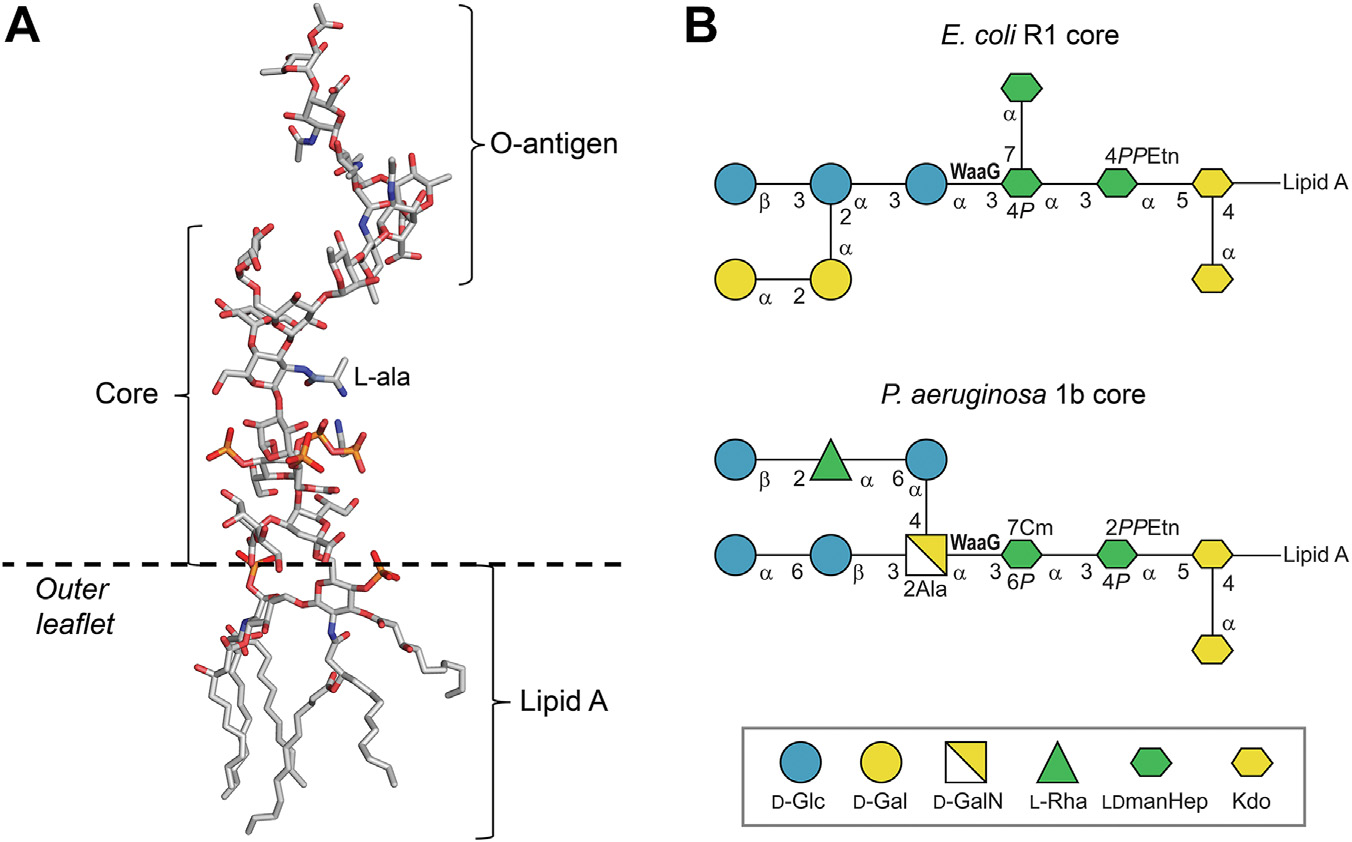
**Lipopolysaccharide (LPS) structure of*****P. aeruginosa***. *A*, overall LPS structure represented by a snapshot from a molecular dynamics simulation of semirough LPS from *P. aeruginosa* serotype O10 containing core glycoform 2 (60); C atoms are colored *gray*, O atoms *red*, N atoms *blue* and P atoms *orange*. Figure produced with PyMOL (v.2.3.3, Schrödinger). *B*, lipopolysaccharide R1 core from *Escherichia coli* (7) and 1b core from *P. aeruginosa* (14) schematically represented using sugar residues in the Symbol Nomenclature for Glycans (SNFG) format (61) drawn by GlycanBuilder2 (62). Monosaccharide codes: d-glucose (d-Glc), d-galactose (d-Gal), d-galactosamine (d-GalN), l-Rhamnose (l-Rha), l-*glycero*-d-*manno*-heptose (ldmanHep), ketodeoxyoctonic acid (Kdo). In *E. coli* the glycosyltransferase WaaG transfers the first hexose residue of the outer core region to a heptose residue in the inner core region.

*E. coli* is the most well-studied Gram-negative bacterium and its LPS is presently classified as having serogroups O1–O188, and together with subgroups, ∼200 O-antigen structures have been determined (6). In contrast to the variable structural features of O-antigenic polysaccharides, only five canonical cores structures have been reported, R1 (7) (Fig. 1*B*), R2, R3, R4, and K12 (8), though like in the polysaccharides, partial substitution patterns make the cores heterogeneous. The substituents that may be nonstoichiometrically present are *O*-acetyl groups and in the cores, in particular, phosphoryl groups.

*Pseudomonas aeruginosa* is another Gram-negative bacterium (9), which is commonly found in environmental reservoirs (*i.e.*, soil, plants, and water). If transmitted to humans it may act as an opportunistic pathogen that causes acute life-threatening infections. For cystic fibrosis patients, who are particularly vulnerable to infections with *P. aeruginosa*, the bacterium triggers a chronic lung infection leading to morbidity and mortality (10, 11). Currently 20 major serotypes, O1 to O20, have been classified for *P. aeruginosa* based on the variable O-antigen polysaccharide part of the LPS according to the International Antigenic Typing Scheme (12, 13) and two different core types have been identified, on the one hand glycoforms 1a and 1b (Fig. 1*B*), and on the other glycoform 2 (14), where it is only the latter that carries an O-antigen polysaccharide.

The core region of the LPS in both *E. coli* and *P. aeruginosa* has branched structures and is subdivided into the inner core consisting of ketodeoxyoctonic and heptose sugar residues and the outer core containing different types of hexoses (Fig. 1*B*). Besides carboxylate groups as part of the ketodeoxyoctonic acid (Kdo) residues, phosphoryl and diphosphoethanolamine groups are present on the heptose residues making the inner core negatively charged. Furthermore, the second heptose residue of the *P. aeruginosa* core contains an unusual substituent, namely, a carbamoyl group attached at position O7, and this heptose is the sugar being glucosylated by the first hexose residue of the outer core. In both *E. coli* and *P. aeruginosa* the substituting residue is an α-(1→3)-linked hexose residue, d-glucose in the former and *N*-(l-alanyl)-d-galactosamine in the latter. Notably, in some strains of *P. aeruginosa* this α-(1→3)-linked residue is *N*-acetyl-d-galactosamine (15, 16), a commonly occurring sugar in bacterial polysaccharides. The hexosyl sugar residue in each of the bacterial species is transferred by a glycosyltransferase (GT) referred to as WaaG to form an α-(1→3)-linkage (12, 17, 18, 19). The LPS core oligosaccharide of *Pseudomonas syringae* pv. *tomato* DC3000 differs to that of glycoforms 1 (Fig. 1*B*) in that instead of the terminal α-(1→6)-linked glucosyl residue it contains a β-(1→2)-linked *N*-acetyl-d-glucosamine residue (20).

Many pathogenic strains of bacteria have developed resistance to antibiotics (21) that are used for treatment of common infections and the prevention of infections during surgery and cancer chemotherapy (22). Small-scaffold antibiotics that are hydrophilic (*i.e.*, β-lactams) can cross the OM through β-barrel porins, but once the scaffold becomes larger than ∼600 Da the molecule cannot. This exclusion limit restricts the passage of antibiotic classes, such as glycopeptides. Many lipophilic antibiotics such as aminocoumarins, macrolides, and rifamycins, are also larger than 600 Da. These antibiotics cannot pass through β-barrel porins or cross the OM by diffusion and are consequently ineffective against Gram-negative bacteria. Large-scaffold antibiotics can be coaxed into the cell once the synthesis of the OM is perturbed. When the biosynthesis of LPS is inhibited by gene knockout, cells often remain viable but they are more permeable to large-scaffold antibiotics (23, 24). Inhibition of LPS biosynthesis is therefore touted as a promising strategy for developing antibiotic adjuvants that could help in combatting resistance to antibiotics (25). The deletion of the gene encoding WaaG in *E. coli* results in an inability to synthesize the outer core and attach the O-antigen polysaccharide of the LPS (termed a deep rough phenotype) (18). The resulting LPS layer also has an 80% reduction in heptose phosphorylation of the inner core oligosaccharide, which is detrimental for the OM stability (26). Furthermore, the inner core phosphates are required both for completion of LPS synthesis and transport of the LPS to the OM in *P. aeruginosa* PAO1 (27). Most importantly, deletion of the gene encoding WaaG causes increased susceptibility to seven classes of antibiotics (23, 24). This latter observation indicates that WaaG is a valid target for inhibition by small molecules.

Our previous studies have focused on identifying small molecules that bind and inhibit WaaG activity in *E. coli* (28, 29, 30). For these studies the crystal structures of WaaG from *E. coli* have been extremely useful as a reference (31). Herein, we present crystal structures of WaaG from *P. aeruginosa* bound to different potential nucleotide-sugar substrates. Based on these results we produced a PaWaaG mutant (PaWaaG-5mut) and investigated the binding of potential substrates and their subsequent hydrolysis for both the WT enzyme and PaWaaG-5mut *via* solution state NMR spectroscopy. These data provide clear insights into the substrate specificity of PaWaaG and will serve as a reference for the future development of inhibitors.

## Results

### Overall structure of *P. aeruginosa WaaG*

**Figure 2:**
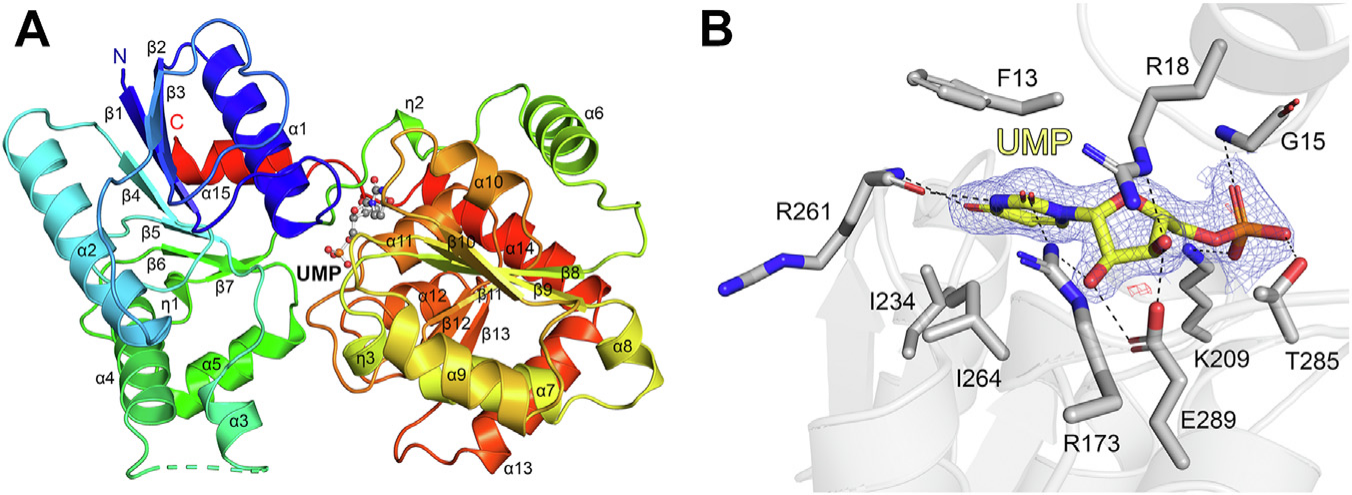
**Crystal structure of the PaWaaG in complex with UMP.***A*, PaWaaG monomer is shown as a rainbow cartoon with secondary structure annotation. UMP is shown as a *ball-and-stick* model. *B*, hydrogen bond network for UMP in the PaWaaG active site. Amino acids involved in ligand coordination are depicted as *sticks*. Hydrogen bond interactions are shown as *dashed lines*. The 2*F*_o_ − *F*_c_ electron density map around UMP is contoured at 1.3 *σ* (*blue*), and the *F*_o_ − *F*_c_ electron density maps are contoured at +3.0 *σ* (*green*) and −3.0 *σ* (*red*). UMP is depicted as a *stick* model; C atoms are colored *yellow*, O atoms *red*, N atoms *blue*, and P atoms *orange*. Figures were produced with PyMOL (v.2.3.3, Schrödinger).

The structure of *P. aeruginosa* WaaG (PaWaaG) in complex with the product analog UMP was solved in the space group *P*2_1_ to 2.0 Å resolution. Data collection and refinement statistics are presented in Table 1. The PaWaaG monomer displays a typical GT-B fold (31, 32, 33), consisting of two “Rossmann-like” β/α/β domains. The N-terminal domain (residues 1–165 and 359–370) is comprised of a seven-stranded twisted β-sheet (β1–7), which is surrounded on both sides by one 3_10_-helix (η1) and six α-helices (α1-5 and α15), with the last α-helix (α15) crossing over from the C terminus to contact the N-terminal domain. The C-terminal domain (residues 170–355) is comprised of a six-stranded twisted β-sheet (β8-13), flanked on both sides by two 3_10_-helix (η2-3) and nine α-helices (α6-14) (Fig. 2*A*). The PaWaaG-UMP structure shows clear density for UMP (Fig. 2*B*), which binds in a deep cleft between the N- and C-terminal domains (Fig. 2*A*). The UMP nucleotide is positioned by three residues from the N-terminal domain and seven residues from the C-terminal domain. Specifically, the uracil base is supported by hydrogen bonding with Arg173 and the backbone nitrogen of Arg261, in addition to hydrophobic interactions with Phe13, Ile234, and Ile264. The ribose moiety is positioned by hydrogen bonds involving Arg173, Arg18, and Glu289. Lastly, the monophosphate of the nucleotide is supported by three hydrogen bonds with Lys209, Thr285, and the backbone nitrogen of Gly15 (Fig. 2*B*).

**Table 1:** X-ray crystallography data collection and refinement statistics

Sample	PaWaaG-UMP	PaWaaG-UDP-glucose	PaWaaG-UDP-galactose	PaWaaG-UDP-GalNAc
Data collection				
PDB code	8B5Q	8B5S	8B62	8B63
Space group	P2_1_	P2_1_	P2_1_	P2_1_
Cell dimensions:				
a, b, c (Å)	41.9, 108.4, 92.4	58.6, 94.4, 70.2	41.9, 109.7, 94.6	41.7, 109.9, 94.3
α, β, γ (°)	90.0, 90.1, 90.0	90.0, 101.4, 90.0	90.0, 90.1, 90.0	90.0, 90.2, 90.0
Resolution (Å)	2.00–108.4 (2.0–2.05)	1.95–57.5 (1.95–2.00)	2.02–47.5 (2.02–2.06)	2.20–54.9 (2.20–2.27)
Total reflections	179,680 (14,280)	182,700 (11,379)	360,581 (17,646)	210,558 (18,837)
Unique reflections	53,974 (4041)	54,511 (3796)	55,384 (2657)	43,178 (3679)
*R*_merge_	0.129 (0.974)	0.080 (0.411)	0.093 (0.757)	0.149 (0.983)
*R*_pim_	0.119 (0.852)	0.073 (0.372)	0.040 (0.309)	0.114 (0.716)
CC_1/2_	0.911 (0.360)	0.979 (0.755)	0.998 (0.788)	0.991 (0.554)
*I*/σ	6.6 (1.4)	10.0 (3.8)	13.5 (2.3)	5.5 (1.4)
Completeness	96.9 (99.7)	99.9 (99.2)	99.0 (95.9)	99.9 (100)
Redundancy	3.3 (3.5)	3.4 (3.0)	6.5 (6.6)	4.9 (5.1)
Refinement				
*R*_work_/*R*_free_ (%)	19.4/24.4	17.9/21.9	17.4/22.0	21.9/25.8
*B*-factors:				
Protein	40.6	18.0	32.9	40.6
Ligand	37.3	18.6	33.6	35.6
Water	41.7	26.5	38.5	36.2
R.m.s. deviations:				
Bond lengths (Å)	0.010	0.003	0.010	0.010
Bond angles (°)	1.59	0.87	1.50	1.53
Ramachandran statistics:				
Favored (%)	100	100	99.7	100
Outliers (%)	0	0	0.3	0

Values in parentheses are for the highest-resolution shell.

### UDP-glucose, UDP-galactose, and UDP-GalNAc binding in PaWaaG

**Figure 3:**
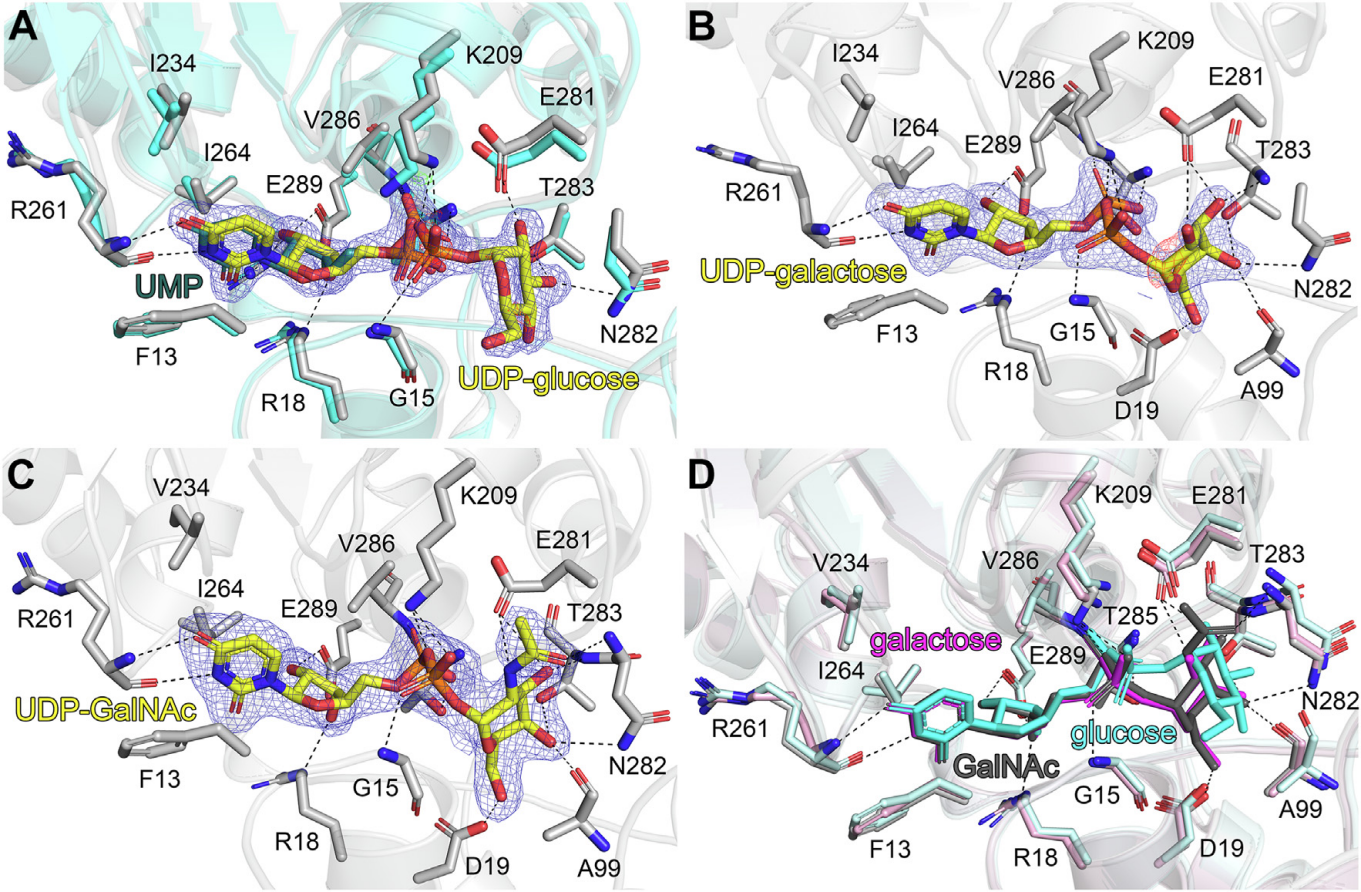
**Comparison of substrate binding in PaWaaG.** Hydrogen bond networks for (*A*) UDP-glucose, (*B*) UDP-galactose, and (*C*) UDP-GalNAc in the PaWaaG active site. In panel *A*, PaWaaG-UDP-glucose (amino acids in light *gray* color) is superimposed with PaWaaG-UMP (amino acids in *cyan* color). In panels (*A–C*) nucleotides are depicted as *stick* models; C atoms are colored *yellow* (UDP-glucose, UDP-galactose, and UDP-GalNAC) or *dark teal* (UMP), O atoms *red*, N atoms *blue*, and P atoms *orange*. Amino acids involved in ligand coordination are depicted as *sticks*. Hydrogen bond interactions are shown as *dashed lines*. The 2*F*_o_ − *F*_c_ electron density maps (*blue*) around the substrates are contoured at 1.0 *σ* (UDP-GalNAc) or 1.3 *σ* (UDP-glucose and UDP-galactose), and the *F*_o_ − *F*_c_ electron density maps are contoured at +3.0 *σ* (*green*) and 3.0 *σ* (*red*). *D*, comparison of UDP-glucose (*cyan*), UDP-galactose (*magenta*) and UDP-GalNAc (*dark gray*) binding in PaWaaG. The hydrogen bond interactions correspond to the PaWaaG-UDP-GalNAc structure. Figures were produced with PyMOL (v.2.3.3, Schrödinger).

In order to gain insights into substrate binding in PaWaaG we solved structures of the enzyme in complex with the nucleotide sugars UDP-glucose, UDP-galactose, and UDP-GalNAc in space group *P*2_1_ to resolutions of 1.95, 2.02, and 2.20 Å, respectively. Data collection and refinement statistics are presented in Table 1. Analysis of the structures showed unambiguous electron density for the three different nucleotide sugars (Fig. 3, *A*–*C*). In each of these structures the asymmetric unit contains two monomers. For the PaWaaG-UDP-galactose structure both monomers have unambiguous density for UDP-galactose. The PaWaaG-UDP-glucose and PaWaaG-UDP-GalNAc structures on the other hand, only have clear electron density for the relevant nucleotide sugars in one monomer and the second monomer is modeled with UDP only. While there was a weak density for the sugar moieties in the second monomer, the quality was not sufficient to position the sugars unambiguously. This may be a result of flexibility in the active site, substrate hydrolysis during crystallization, or partial substrate degradation prior to crystallization during freezing/thawing of the nucleotide sugars. Comparison of the structures (including PaWaaG-UMP) indicated no significant overall conformational changes, with low RMSD values ranging from 0.252 to 0.552 Å for all structural comparisons. Inspection of the substrate-binding site shows that the UDP component of the nucleotide sugars superimposes well with UMP from the PaWaaG-UMP structure, as exemplified with PaWaaG-UDP-glucose (Fig. 3*A*). Furthermore, the active site residues which aid in positioning either UMP or UDP occupy the same positions in all of the structures (Fig. 3, *A*–*C*).

However, comparison of the sugar moieties in the structures indicates several important differences. In PaWaaG-UDP-glucose there are three hydrogen bond interactions (Glu281, Thr283, and Asn282) which position glucose (Fig. 3*A*), whereas there are two additional hydrogen bond interactions (Asp19 and the backbone nitrogen of Ala99) which orient galactose and GalNAc in the PaWaaG-UDP-galactose and PaWaaG-UDP-GalNAc structures (Fig. 3, *B* and *C*), as well as the side-chain nitrogen atom of Asn282 which is in hydrogen bonding distance (3.1 Å) to O4′ of Gal and GalNAc. Interestingly, structural comparisons show that, while the galactose portion of PaWaaG-UDP-galactose superimposes perfectly with the galactose component of GalNAc from PaWaaG-UDP-GalNAc, it occupies a quite different position to the glucose moiety of PaWaaG-UDP-glucose (Fig. 3*D*). Notably, whereas the dihedral angle *ω* (O5-C5-C-6-O6) of the exo-cyclic hydroxymethyl group in the glucosyl residue in PaWaaG-UDP-glucose has the *gauche-trans* conformation with *ω* = 79°, that is, the same conformation as is the EcWaaG-UDP-2F-glucose structure (Protein Data Bank [PDB] ID: 2iw1) with *ω* = 55°, which is one of the two highly populated conformations of *ω* in glucose in solution (34), the dihedral angle *ω* in the galactose residues in PaWaaG-UDP-galactose and PaWaaG-UDP-GalNAc have the *gauche-gauche* conformation with *ω* = −29° and *ω* = −32°, respectively, a conformation only sparsely populated in solution (35).

### Comparison of *P. aeruginosa* and *E. coli WaaG* structures

**Figure 4:**
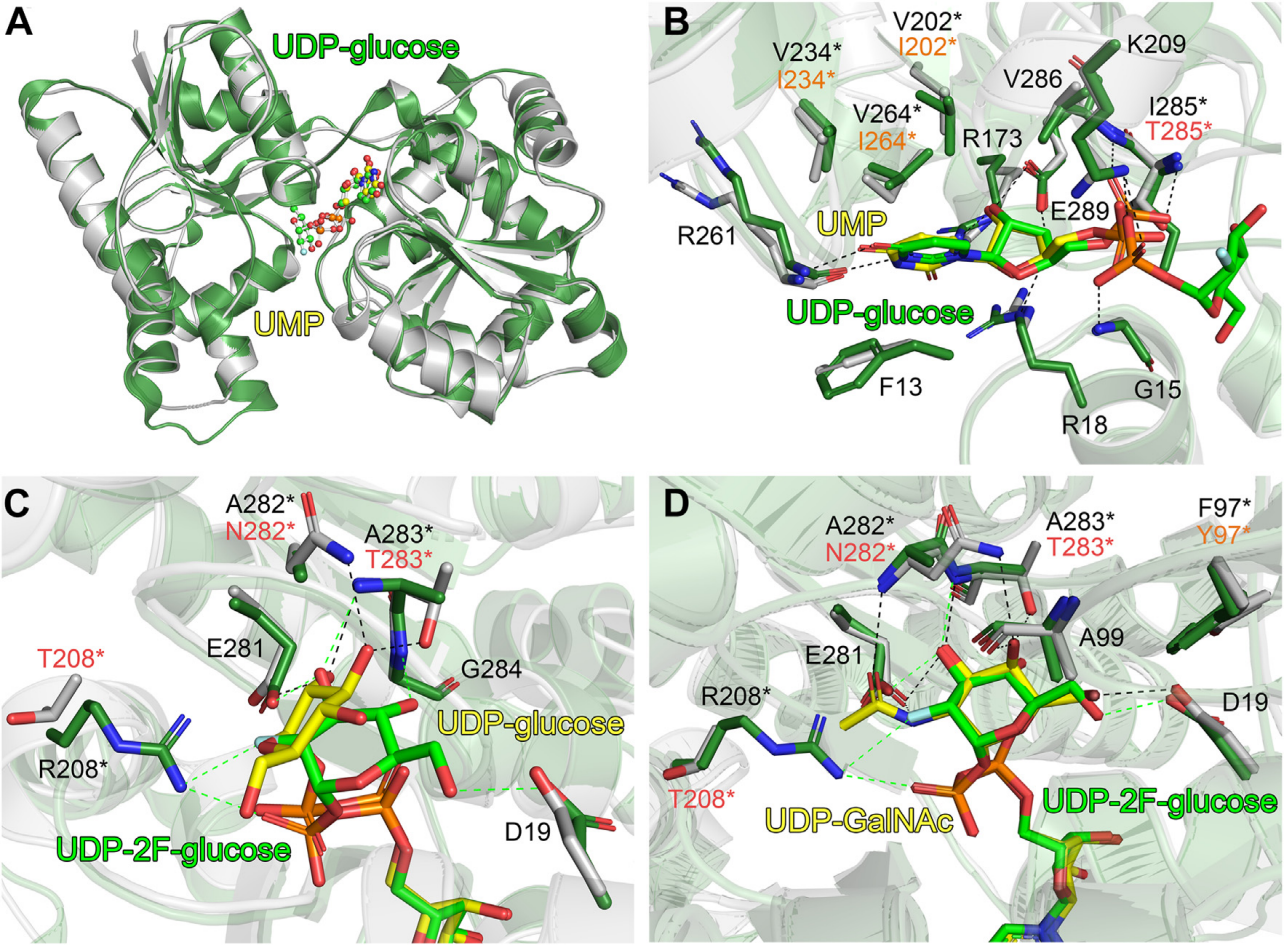
**Comparison of PaWaaG structures with EcWaaG.***A*, superposition of PaWaaG-UMP (*gray*) and EcWaaG-UDP-2F-glucose (*dark green*, PDB ID: 2iw1) monomers. Nucleotides are depicted as *ball-and-stick* models; C atoms are colored *yellow* (UMP) or *green* (UDP-2F-glucose), O atoms *red*, N atoms *blue*, and P atoms *orange*. Comparison of EcWaaG UDP-2F-glucose binding with (*B*) UMP, (*C*) UDP-glucose and (*D*) UDP-GalNAc binding in PaWaaG. Hydrogen bond interactions for the EcWaaG and PaWaaG complexes are shown as *green* and *black dashed lines*, respectively. Amino acids involved in ligand coordination are depicted as *sticks* and numbering refers to the EcWaaG complex. Where coordinating amino acids are not conserved between the structures the relevant residue from PaWaaG is labeled, with *red* and *orange* text referring to nonconserved and physiochemically conserved differences, respectively. Figures were produced with PyMOL (v.2.3.3, Schrödinger).

A structural similarity search was performed using the DALI web server (36), which indicated that PaWaaG was most structurally similar to *E. coli* WaaG GT (EcWaaG), with which it shares 50 % amino acid sequence identity. Comparison of all four PaWaaG structures with EcWaaG in complex with UDP-2-deoxy-2-fluoro-D-glucose (PDB ID: 2iw1, (31)) indicated no significant overall conformational differences (Fig. 4*A*), with low RMSD values ranging from 0.843 to 1.013 Å for all structural comparisons. Superposition of PaWaaG-UMP with EcWaaG-UDP-2F-glucose showed the nucleotide component of the ligands to occupy the same position within the active site (Fig. 4*B*). Amino acid residues that support the nucleotide though hydrogen bonding were shown to be entirely conserved, whereas there were some differences in a small hydrophobic area in the binding pocket in close proximity to the uracil base. Specifically, Ile202, Ile234, and Ile264 (PaWaaG numbering) are all valine amino acids in EcWaaG (Figs. 4*B* and S5). However, these differences are unlikely to be significant for nucleotide-sugar substrate binding as valine and isoleucine are both hydrophobic amino acids which only differ by the extension of a single methylene group, and they therefore have similar chemical properties.

An interesting point of difference between the structures is the large difference in the binding modes of the glucose and 2F-glucose sugar moieties (Fig. 4*B*). In PaWaaG the glucose group is twisted significantly relative to 2F-glucose in EcWaaG, and is supported by hydrogen bonding with the sidechains of Glu281, Asn282, and Thr283 (Fig. 4*C*). 2F-glucose in EcWaaG is also positioned by hydrogen bonding with the sidechain of Glu281; however, the rest of the hydrogen bond network is entirely different. Specifically, the 2F-glucose sugar hydrogen bonds with the mainchain nitrogen atoms of Ala283 (Thr283 in PaWaaG) and Gly284 (also glycine in PaWaaG), in addition to the sidechains of Asp19 (also aspartate in PaWaaG) and Arg208 (Thr208 in PaWaaG) (Fig. 4*C*). In contrast to the glucose sugar comparisons, superposition of PaWaaG-UDP-galactose and PaWaaG-UDP-GalNAc with EcWaaG-UDP-2F-glucose revealed that the twisted conformation observed for glucose in PaWaaG-UDP-glucose relative to the EcWaaG structure is not present. Instead, galactose and GalNAc adopt the same overall position and orientation as 2F-glucose (Fig. 4*D*). The hydrogen bond networks for galactose and GalNAc are also very similar to 2F-glucose in EcWaaG. The main differences between the structures are that the sidechains of Asn282 and Thr283 in PaWaaG which interact with the sugar are not observed in EcWaaG, where the equivalent residues are both alanines (Ala282 and Ala283). The interaction between the main-chain oxygen of Ala99 and the hydroxyl (OH) group at the C4′ position of each UDP-hexose bound to PaWaaG is also not observed in EcWaaG, since the OH group of 2F-glucose has the opposite stereochemistry to what is observed for galactose, and therefore points away from rather than toward these amino acids. In addition, the hydrogen bond between R208 and 2F-glucose in EcWaaG is not present for PaWaaG where the equivalent residue is a threonine (Thr208), the side-chain of which is too short to be within hydrogen bonding range to the galactose or GalNAc (Fig. 4*D*).

### Production of PaWaaG-5mut protein

**Figure 5:**
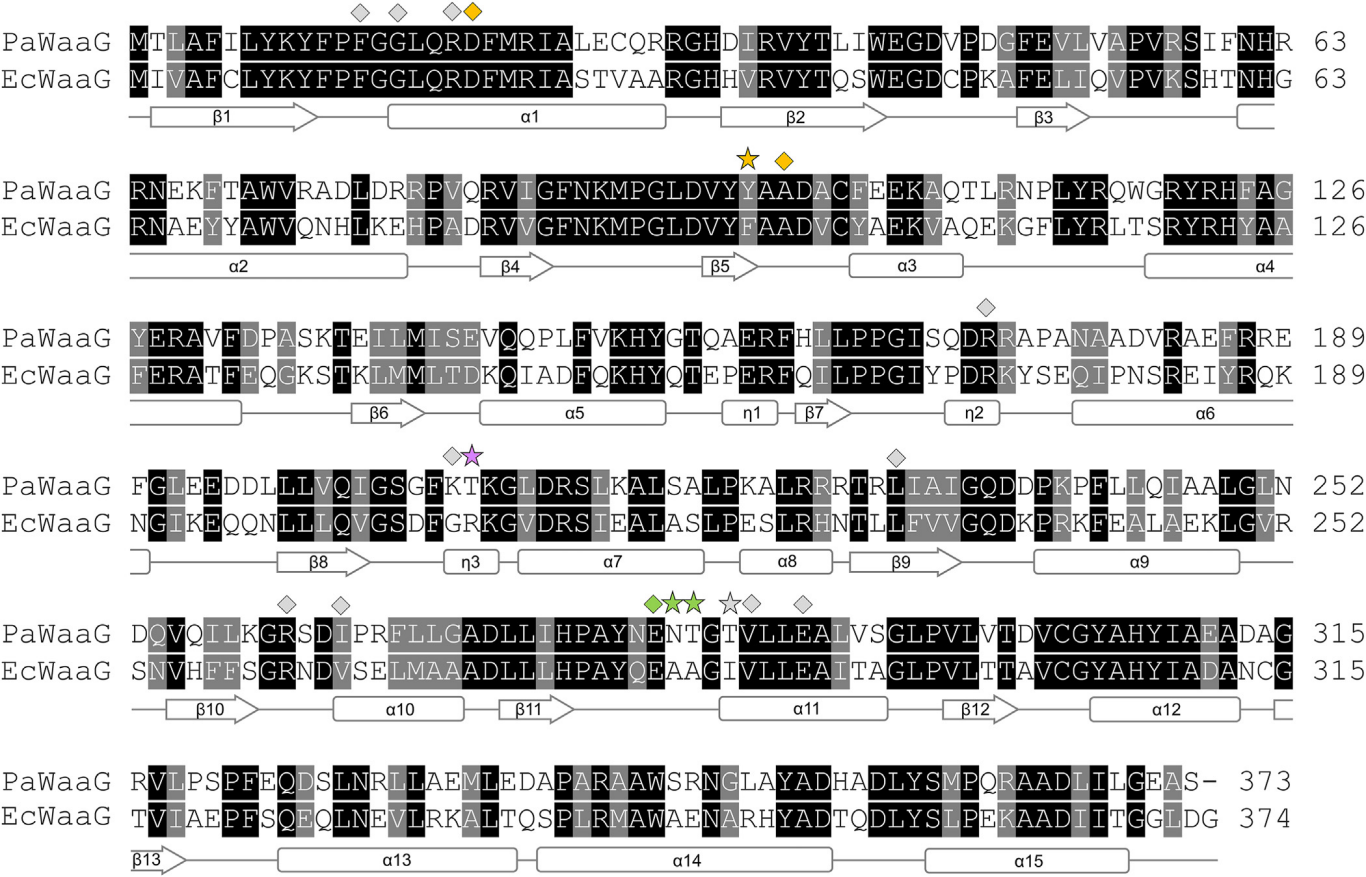
**Structure-based sequence alignment of *P. aeruginosa* WaaG (UniProt:****Q9HUF6****) and *Escherichia coli* WaaG (UniProt:****B7L754****) performed using Clustal Omega through theEuropean Bioinformatics Institute (EBI)webserver.** The resulting alignment is colored according to sequence similarity using BOXSHADE. Identical residues are shaded *black*, while *dark gray* shading indicates amino acids with conserved physicochemical properties. Residues important for the positioning of nucleotide sugars in PaWaaG are shown above the alignment. *Light gray* symbols indicate UMP/UDP interacting amino acids. *Green* symbols show residues that position the glucose, galactose, and GalNAc moieties of the nucleotide sugars. *Yellow* symbols indicate residues that position galactose and GalNAc only. The *purple* symbol indicates a residue (Thr208, PaWaaG numbering) that is proposed to be important for the specificity of PaWaaG toward UDP-GalNAc. *Stars* indicate five amino acid which were mutated in the PaWaaG sequence to the equivalent EcWaaG residues to produce the PaWaaG-5mut mutant. The secondary structure corresponding to the amino acid sequence of PaWaaG is displayed below the alignment.

**Figure 6:**
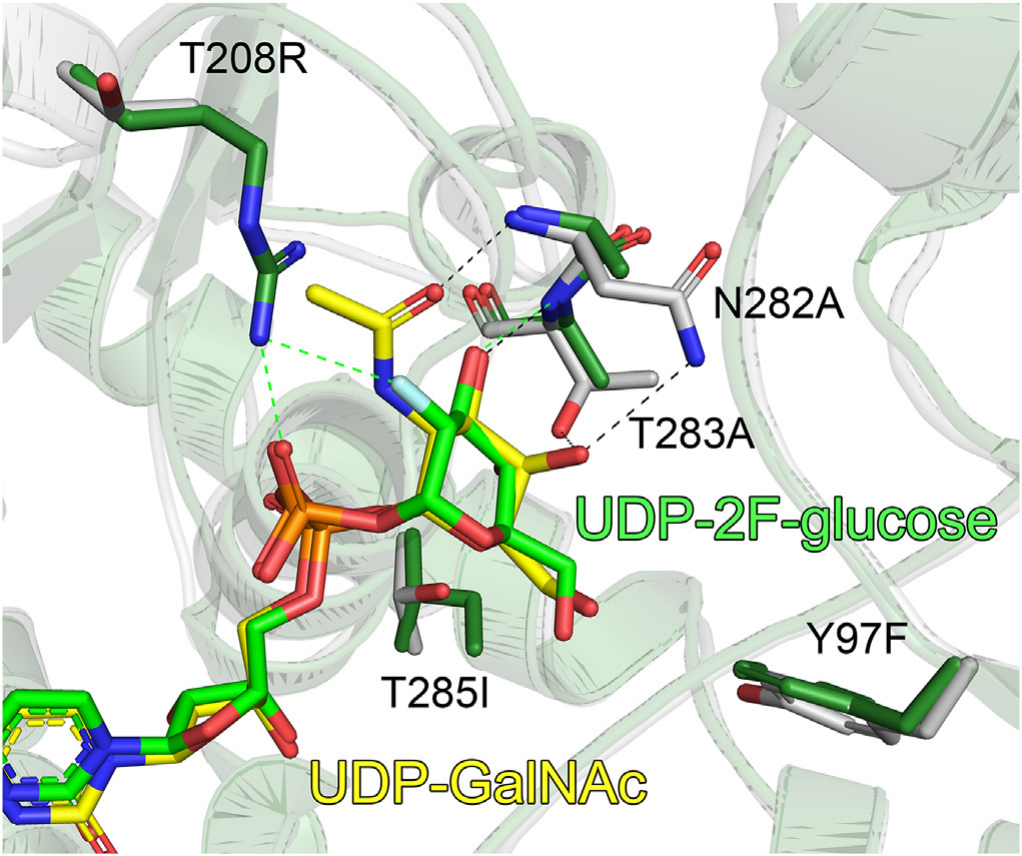
**Important residues in the PaWaaG-5mut construct.** Superposition of PaWaaG-UDP-GalNAc (*gray*) and EcWaaG-UDP-2F-glucose (*dark green*, PDB ID: 2iw1) monomers. Nucleotides are depicted as *stick models*; C atoms are colored *yellow* (UDP-GalNAc) or *green* (UDP-2F-glucose), O atoms *red*, N atoms *blue*, and P atoms *orange*. Hydrogen bond interactions for the EcWaaG and PaWaaG complexes are shown as *green* and *black dashed lines*, respectively. The five mutations in the PaWaaG-5mut construct change those amino acids to the equivalent amino acids present in EcWaaG. Figures were produced with PyMOL (v.2.3.3, Schrödinger). PDB, Protein Data Bank.

EcWaaG has been extensively studied and is known to specifically transfer glucose from UDP-glucose to the l-glycero-d-*manno*-heptose-II (HepII) residue of the inner core of the LPS (18, 37). Importantly, structural information for EcWaaG in complex with its endogenous substrate UDP-glucose is also available (31). Comparison of our PaWaaG complexes with EcWaaG-UDP-2F-glucose showed a high degree of structural conservation in the active site but also revealed interesting differences in regards to how the different nucleotide sugars interact with the protein. Analysis of the region of the binding pocket which positions the sugar moieties of the compounds shows five key amino acid differences between PaWaaG and EcWaaG (Figs. 5 and 6). We therefore decided to design a PaWaaG mutant (named PaWaaG-5mut) where the residues were changed to those of the *E. coli* protein and we tested whether the PaWaaG-5mut (Y97F/T208R/N282A/T283A/T285I) would behave similarly to EcWaaG, as it would have a different donor substrate specificity compared to the WT PaWaaG enzyme. The PaWaaG-5mut construct was expressed and purified in an identical manner to what was performed for the WT PaWaaG protein (refer to Experimental procedures section).

### NMR analysis of substrate specificity

**Figure 7:**
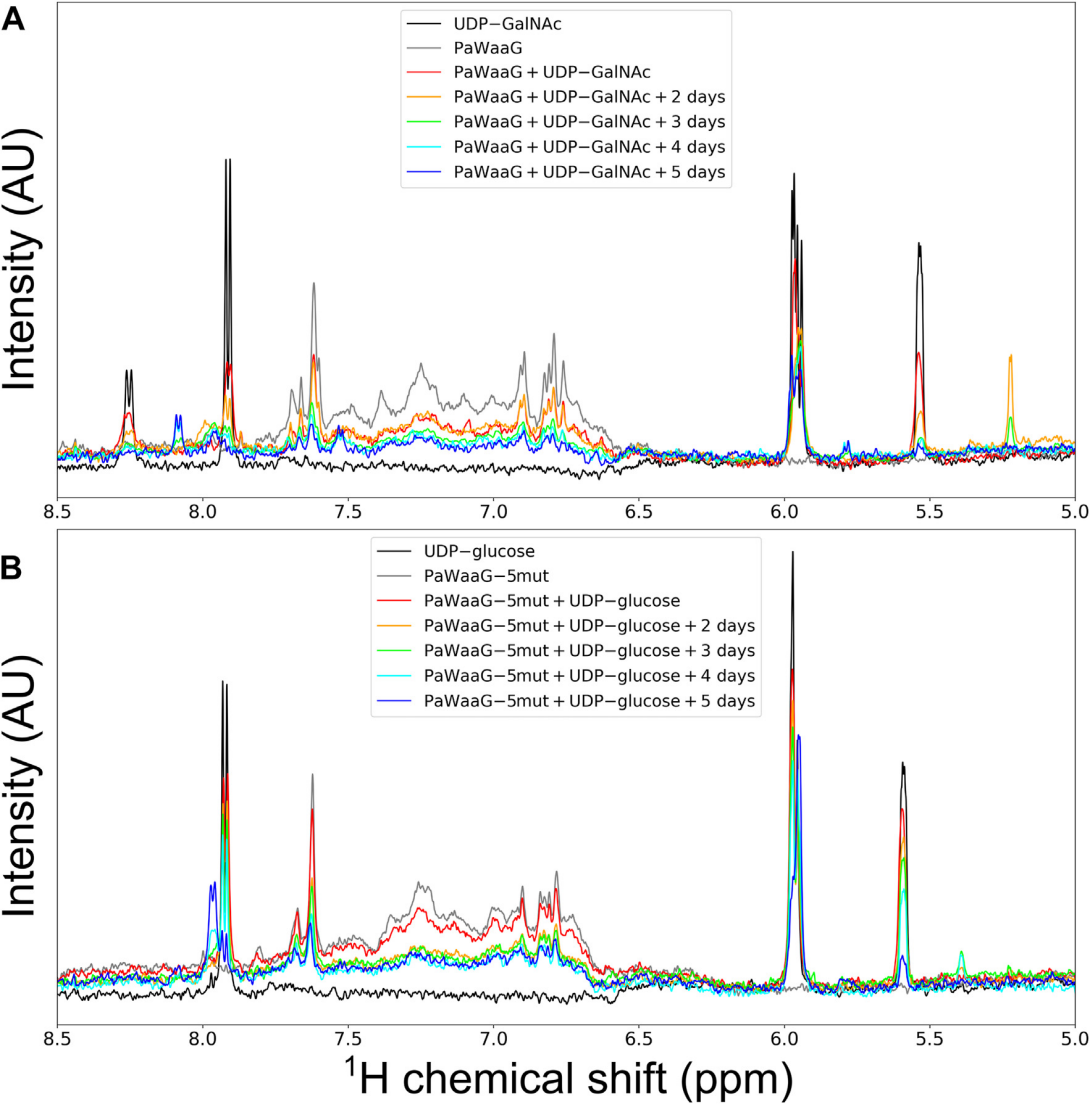
**Solution NMR experiments of PaWaaG with potential donor sugar substrates.** Hydrolysis of (*A*) UDP-GalNAc by PaWaaG and (*B*) UDP-glucose by PaWaaG-5mut. The panels display the downfield part of the ^1^H NMR spectrum where deshielded protons (*i.e.*, hydrogens in protein amide and aromatic groups, hydrogens close to electronegative atoms in sugars and nucleotides) have chemical shifts. Spectra of proteins together with sugar donors are color coded according to the number of days after substrates were added. Spectra of UDP-GalNAc or UDP-glucose in buffer only (*black*) and of proteins before the addition of substrate (*gray*) are also shown.

In lieu of a GT assay, which is possible when a rough acceptor LPS is available (38) we utilized solution NMR to study the hydrolysis of potential nucleotide-sugar substrates, to reveal which of the sugar donors would be the most likely substrate for WT PaWaaG and PaWaaG-5mut. Experiments were performed where the hydrolysis of three potential substrates (UDP-glucose, UDP-galactose, and UDP-GalNAc) was monitored over 5 days. A glucose-containing analog of UDP-GalNAc, UDP-GlcNAc, was also included in the experiments, so that all combinations of substrate moieties could be compared. Naturally, the reaction with the respective native acceptor LPS cores are expected to have dramatically higher turnover. Control experiments to verify that autohydrolysis did not occur were also performed, and these demonstrated that none of the substrates were hydrolyzed in the absence of enzyme. Therefore, it is reasonable to believe that if a potential donor substrate could be hydrolyzed in the presence of PaWaaG, it is likely a true substrate. Similar hydrolysis experiments have previously been performed for *E. coli* WaaG (39). Significant hydrolysis of UDP-GalNAc by PaWaaG and hydrolysis of UDP-glucose by PaWaaG-5mut was observed (Fig. 7), while no hydrolysis of other putative donor substrates was observed for either protein, indicating a preference for UDP-GalNAc by PaWaaG, while the mutant designed to mimic *E. coli* WaaG indeed has a preference for UDP-glucose.

The immediate, large decrease in the peak heights, for example, at 5.54 or 5.60 ppm for H1’’ of the hexose group in UDP-GlcNAc and UDP-glucose, respectively, in the presence of protein, as compared to in only buffer, indicate that not only do interactions between the proteins and substrates take place but that the substrates are in fact hydrolyzed. Subsequent hydrolysis is slow and needs days to achieve a couple of turnovers per protein (initial sugar donor-to-protein ratios were around 5:1 for both PaWaaG and PaWaaG-5mut). The hydrolysis reactions of UDP-GalNAc and UDP-glucose result in hexoses, for which the resonances of their anomeric protons, α-anomeric form, can be identified at ∼5.2 ppm (40) and disappearance of H1′ resonances of UDP-hexose at ∼5.6 ppm (28). Furthermore, downfield ^1^H NMR chemical shift displacements occur for H6 of the uridine residue upon hydrolysis to UDP (28). The signals from both PaWaaG and PaWaaG-5mut decrease during the first few days, including the sharp peaks observed at around 7.6 ppm, indicating precipitation, but level off later on. The most likely explanation for this is that the initial concentration of PaWaaG was over the solubility limit. The hydrolysis of UDP-GalNAc and UDP-glucose can also be detected by phosphorus NMR spectra (Fig. S1, *A* and *B*) where new peaks appear at −3.1 and −7.6 ppm, arising from the distal P_β_ and proximal P_α_ atoms in the resulting UDP molecule (41) when the hexose has been cleaved off.

Significant hydrolysis of the putative substrates, UDP-glucose and UDP-galactose, by PaWaaG was not detected (Figs. S1, *C* and *D* and S2, *A* and *B*), indicating that the WT enzyme is only able to hydrolyze UDP-GalNAc specifically. Conversely, PaWaaG-5mut did not hydrolyze UDP-GalNAc or UDP-GlcNAc (Figs. S1, *E* and *F* and S2, *C* and *D*). Moreover, ^1^H NMR line-broadening of resonances from sugar donors when PaWaaG was present indicate that the WT protein also interacts with UDP-glucose and UDP-galactose, but that this interaction does not result in a hydrolysis reaction (Fig. S2, *A* and *B*). In contrast, for PaWaaG-5mut there is no or only a slight line broadening of UDP-GalNAc or of UDP-GlcNAc, indicating that PaWaaG-Mut5 is more specific for UDP-glucose. Overall these results demonstrate that of the substrates tested, WT PaWaaG was only able to hydrolyze UDP-GalNAc, whereas the engineered variant PaWaaG-5mut only hydrolyzed UDP-glucose.

### PaWaaG cannot complement the phenotype of an *E. coli ΔwaaG* strain

**Figure 8:**
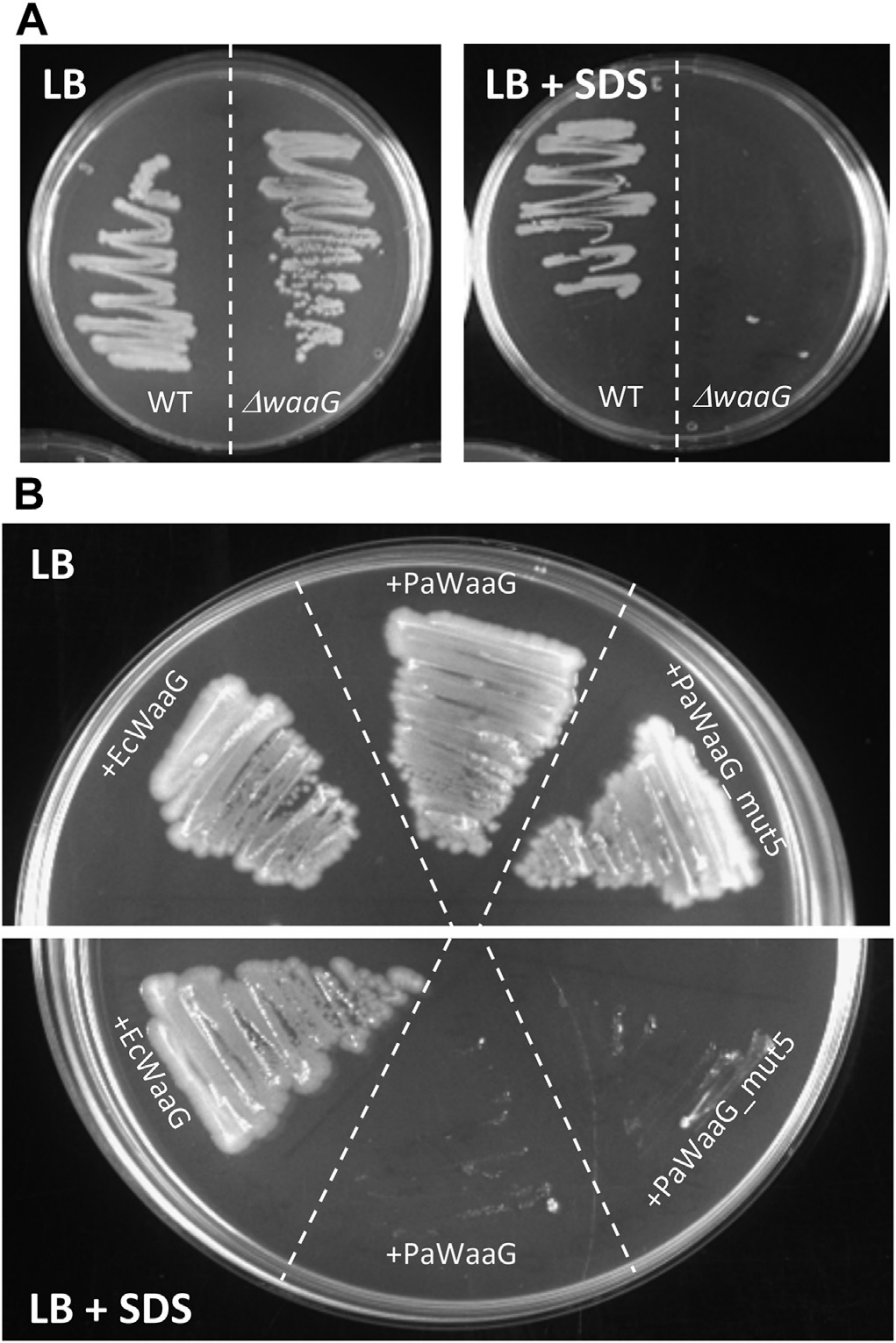
**Complementation of the phenotype in an *Escherichia coli* strain lacking WaaG (Δ*waaG*).***A*, the *E. coli* Δ*waaG* strain grows comparably to a WT *E. coli* strain on LB agar (*leftpanel*) but is sensitive to SDS (*rightpanel*). *B*, EcWaaG, PaWaaG, and PaWaaG-5mut were expressed in *E. coli* Δ*waaG* and plated on LB agar (*top panel*) or LB agar with 0.0625% (w/v) SDS (*bottom panel*). Plates contained 100 μg/ml ampicillin. Proteins were expressed at low levels by the inclusion of 0.02% (w/v) glucose in the agar.

The X-ray crystal structures of PaWaaG with bound nucleotide-sugars together with our NMR analysis of substrate specificity suggests that PaWaaG is functionally different from the EcWaaG homolog. We therefore decided to determine whether PaWaaG and PaWaaG-5mut were capable of complementing the phenotype of an *E. coli* strain lacking the *waaG* gene. The *E. coli* Δ*waaG* strain is a deep rough mutant where the LPS is truncated after HepII. It grows comparably to WT *E. coli* on LB agar but is sensitive to low concentrations of SDS (Fig. 8*A*), a result consistent with the study of Yethon *et al.* (18). We utilized the SDS sensitive phenotype to establish a functional assay. EcWaaG, PaWaaG, and PaWaaG-5mut were expressed in *E. coli* Δ*waaG* and the strains were plated on LB agar with and without 0.0625% (w/v) SDS. All strains were viable; however, only the strain expressing EcWaaG was viable in the presence of 0.0625% (w/v) SDS. Overall, this indicates that EcWaaG can complement the SDS-sensitive phenotype of the *E. coli* Δ*waaG* strain, whereas PaWaaG and PaWaaG-5mut cannot (Fig. 8).

## Discussion

Herein, we have determined structures of PaWaaG bound to the putative substrates UDP-glucose, UDP-galactose, and UDP-GalNAc. Comparison of PaWaaG structures with EcWaaG-UDP-2F-glucose indicated that the UDPs superimposed very well and the nucleotide coordinating amino acids residues were highly conserved. The only differences were three residues in a hydrophobic part of the pocket near the uracil base, which are isoleucines in PaWaaG and valines in EcWaaG. These amino acid residues represent conservative changes as both isoleucine and valine are hydrophobic amino acids, differing only by the extension of a single methylene group, suggesting that hydrophobicity is important for this area of the UDP-binding pocket. Strong conservation of the UDP-binding pocket makes evolutionary sense, as nonconserved mutations might perturb overall substrate binding, which would render the enzyme nonfunctional.

Interestingly, while the ring carbon atoms of the galactose and GalNAc sugars superimposed nicely with those of glucose in EcWaaG, the glucose moiety in PaWaaG adopted a very different position. As EcWaaG specifically hydrolyzes UDP-glucose, the EcWaaG-UDP-2F-glucose structure represents productive substrate binding (31) and we therefore speculated that our PaWaaG-UDP-glucose structure indicates nonproductive substrate binding. Solution state NMR experiments revealed that PaWaaG specifically hydrolyzes UDP-GalNAc and that the enzyme binds UDP-glucose but does not hydrolyze it. This indicates that our UDP-GalNAc structure represents productive substrate binding and confirms that the PaWaaG-UDP-glucose structure shows a nonproductive substrate binding mode. It is possible that UDP-glucose may act as a competitive inhibitor of PaWaaG; however, verifying this would require the future development of a PaWaaG GT assay. Interestingly, PaWaaG was also unable to hydrolyze UDP-galactose, despite the crystal structure showing that the sugar binds in a very similar way to GalNAc. This may be due to galactose potentially being quite mobile, as evidenced by the significant *F*_o_-*F*_c_ peak around the anomeric carbon of the sugar, which is not observed in our PaWaaG-UDP-GalNAc structure. This may indicate that the position of GalNAc is more stable, which may provide a more favorable environment for nucleophilic attack on the substrate.

A limitation of our solution NMR experiments is that they only measure donor substrate hydrolysis, and while this can indicate whether a donor sugar is correctly bound and accessible to a water molecule that is activated as a nucleophile, it does not measure *in vivo* GT activity. A GT assay for EcWaaG has been published (29); however, it is not immediately adaptable to PaWaaG as the acceptor substrate is different for the two enzymes (Fig. 1*B*). The future development of a PaWaaG GT assay would require making a Δ*waaG* knockout strain of *Pseudomonas* and then extracting truncated LPS to be used as an acceptor substrate. Such an assay would be particularly useful for structure-based drug design studies targeting PaWaaG, which in addition to X-ray crystal structural information, also requires a means of measuring enzyme inhibition in order for starting compounds to be efficiently developed into potent inhibitors. Nevertheless, the results clearly demonstrating that PaWaaG is only capable of binding one putative substrate, UDP-GalNAc, in a productive way leading to hydrolysis strongly argues that this is the correct donor substrate.

There were five notable differences between PaWaaG and EcWaaG in the sugar binding pocket. We expressed and purified PaWaaG-5mut, designed to resemble the EcWaaG sugar binding site. Solution state NMR confirmed PaWaaG-5mut had a different donor substrate specificity to WT PaWaaG, as it could not hydrolyze UDP-GalNAc and was instead specific for UDP-glucose like EcWaaG. This indicates that the amino acids are the determinants of substrate specificity, which is further explained from a structural perspective. In EcWaaG Arg208 is important for hydrogen bonding to glucose, while in PaWaaG the equivalent residue is Thr208. As threonine has a much shorter side-chain than arginine, it is not within hydrogen bonding range of the sugar. This difference also explains why EcWaaG cannot hydrolyze UDP-GalNAc, as the much longer arginine side-chain would directly clash with the *N*-acetyl group of the substrate. This is further supported by the fact that PaWaaG-5mut can hydrolyze UDP-glucose, but cannot hydrolyze UDP-GlcNAc.

In PaWaaG, Asn282 and Thr283 are important for binding to UDP-galactose and also interfere with binding to UDP-glucose. The side-chains of Asn282 and Thr283 hydrogen bond with the OH group at the C4′ carbon, an interaction absent in EcWaaG where both residues are alanine, which has a small methyl group side-chain incapable of hydrogen bonding. This may result in galactose interacting less tightly with EcWaaG. Furthermore, Thr283 may interfere with UDP-glucose binding in PaWaaG as a threonine would be too close to the aforementioned OH group, as opposed to EcWaaG which has amuch smaller alanine at this position. The structural importance of Tyr97 and Thr285 for PaWaaG substrate specificity is less clear, as they are slightly further away from the sugar moieties compared to the other amino acids, and they are not required for hydrogen bonding with glucose or GalNAc. In EcWaaG the equivalent residues are Ile285 and Phe97, which are both hydrophobic amino acids. This may give this part of the binding pocket in EcWaaG a more hydrophobic character than the PaWaaG, where the corresponding residues both contain OH groups in their sidechains. Further mutational studies would be required to assess the importance of Tyr97 and Thr285 in substrate specificity.

The α-d-Gal*p*NAc-(1→3)-l-α-d-Hep*p* motif (15, 16) is, to the best of our knowledge, only present in the LPS of bacteria in the genus *Pseudomonas*. An amino acid sequence alignment of PaWaaG with homologs from *Pseudomonas putida*, *Pseudomonas fluorescens*, *P. syringae*, and *Pseudomonas cichorii* shows that the PaWaaG amino acids Y97, T208, N282, T283, and T285 are entirely conserved between the species, further supporting that they are required for UDP-GalNAc specificity (Fig. S3) and LPS from *P. aeruginosa* should have the α-d-Gal*p*NAc-(1→3)-l-α-d-Hep*p* or α-d-Gal*p*N(l-Ala)-(1→3)-l-α-d-Hep*p* motifs. A sequence alignment of PaWaaG with homologs from *E. coli*, *Erwinia carotovora*, *Hafnia alvei*, and *Citrobacter freundii*, which have a α-d-Glc*p*-(1→3)-l-α-d-Hep*p* motif in their LPS (and therefore likely use UDP-glucose as a substrate) indicates that PaWaaG residues T208, T283, and T285 are always arginine, alanine, and isoleucine, respectively, in the other proteins, further underscoring their importance (Fig. S4). PaWaaG Asn282, which is important for binding to the *N*-acetyl-d-galactosamine moiety of UDP-GalNAc, is most often alanine in the other bacteria with the exception of *H. alvei* WaaG, which has a serine. This difference is likely tolerated since the side-chain of serine is shorter than asparagine and thus is still too far away to hydrogen bond with the OH at the C4′ atom of GalNAc. In contrast, PaWaaG Y97 is unlikely to be important for the enzymes’ specificity toward UDP-GalNAc as *E. carotovora* and *H. alvei* WaaGs also have a tyrosine at this position (Fig. S4).

Neither PaWaaG or PaWaaG-5mut could complement an *E. coli* Δ*waaG* strain *in vivo*, indicating that they are functionally different to EcWaaG and the UDP-glucose hydrolysis of PaWaaG-5mut alone is insufficient to build the *E. coli* inner core. In addition to hydrolyzing the nucleotide sugar, WaaG also has to transfer it to a heptose in the inner core and so the surface of the protein is very likely in contact with the LPS, at least transiently, while this is happening. Comparison of the LPS cores of *P. aeruginosa* and *E. coli* indicates that the heptose sugar to which d-glucose (EcWaaG) or d-Gal*p*NAc/d-Gal*p*N(l-Ala) (PaWaaG) is added are modified differently. In particular, heptose-II in the *P. aeruginosa* LPS core is phosphorylated at O6 positioned exo-cyclically to the pyranose ring of the sugar residue, whereas in *E. coli* this sugar is phosphorylated at O4 adjacent to the substitution position (O3) of the pyranose ring (Fig. 1*B*). The surface charge of WaaG and amino acid residues which contribute to the LPS-binding site may therefore be important for the overall biological function of the enzymes. While the tertiary structures of EcWaaG and PaWaaG are highly conserved, there are many differences, predominantly located on the surfaces of the proteins (Fig. S5). This results in significant surface charge differences for PaWaaG and EcWaaG (Fig. S6). Furthermore, there are several differences in the likely acceptor LPS binding site between PaWaaG and EcWaaG (Fig. S5) that may be relevant for interaction with the differently phosphorylated core LPS structures. In EcWaaG residue R208, which is important for glucose specificity, may also play a role in phosphate binding. A structural analysis of phosphate binding sites by Copley *et al.* showed that the most common amino acids in α-helix type binding sites in order of frequency are glycine, arginine, threonine, serine, and lysine, whereas the most common amino acids at nonhelix binding sites (*i.e.*, loop regions) are arginine, tyrosine, histidine, lysine, and serine (42). There are ten differences between PaWaaG and EcWaaG in the potential acceptor LPS binding site, of which six amino acids do not have conserved chemical properties (Fig. 5). In each structure there are three amino acids known to be abundant in phosphate binding sites, namely, Ser144, Lys207, and Thr208 in PaWaaG and Lys146, Gly207, and Arg208 in EcWaaG. Taken altogether, the differences in core LPS structure between *P. aeruginosa* and *E. coli* as well as differences in the likely acceptor LPS binding site of the enzymes may be why PaWaaG-5mut was unable to complement *E. coli* Δ*waaG*.

Hitherto, only two species of *Pseudomonas* incorporate α-d-Gal*p*NAc-(1→3)-l-α-d-Hep*p* in their LPS (15, 16) and in most cases, the GalNAc is actually modified further to GalN(l-Ala) (12, 14). This addition resulting in the exposure of an amino group is reminiscent of having glucosamine residues (devoid of their *N*-acetyl groups) in the core region of *Brucella melitensis* (43) leading to a core that has a lower negative charge, which may aid in evading the immune system of the host, in particular to become less sensitive to cationic host defense peptides, also known as antimicrobial peptides (44, 45). We have shown that PaWaaG hydrolyzes UDP-GalNAc; however, the cores of *P. aeruginosa* LPS often contain GalN(l-Ala). There are two possibilities for how this may occur in the cell: (i) WaaG hydrolyzes UDP-GalN(l-Ala) and adds the modified sugar to heptose in the LPS. Examination of our PaWaaG structure confirms there is sufficient space for GalN(l-Ala) in the sugar binding pocket. However, it is unclear how UDP-GalN(l-Ala) is produced by *Pseudomonas in vivo*. (ii) WaaG utilizes UDP-GalNAc and transfers the sugar to the inner core, after which GalNAc is further modified in the LPS to GalN(l-Ala). In this scenario, GalNAc would first need to be *N*-deacetylated before l-Ala could be added. Furthermore, it is known that deacetylation occurs in other processes required for LPS biosynthesis; for example, during Lipid A synthesis, LpxC removes an *N*-acetyl group after which the *N*-acyltransferase LpxD adds a long amide-linked fatty acid to the molecule (46). Interestingly, the gene cluster responsible for synthesis of the LPS core in *P. aeruginosa* PAO1, to which *waaG* belongs (gene locus PA5010), also contains a putative de-*N*-acetylase called *dnpA* (gene locus PA5002) (20, 47). The *dnpA* gene has been shown to be associated with *P. aeruginosa* persistence (48) and resistance to fluoroquinolones (49). It has been proposed that *dnpA* is involved in minor modifications to the LPS; however, the substrate of the enzyme remains unknown. This is consistent with *P. aeruginosa* and *E. coli* Δ*dnpA* deletion strains (48, 50), which show that LPS synthesis is unaffected and the core structure remains intact. We therefore speculate that after PaWaaG has transferred GalNAc to the core LPS, the sugar may be *N*-deacetylated by DnpA, after which an unknown ligase or transferase adds l-Ala, generating GalN(l-Ala).

In conclusion, we present structures of PaWaaG in complex with different UDP nucleotide-sugars and solution state NMR substrate specificity analysis for both the WT enzyme and the PaWaaG-5mut mutant. This analysis revealed the structural determinants for the differences in donor substrate specificity observed between *P. aeruginosa* and *E. coli* WaaG GTs. Interestingly, while PaWaaG-5mut variant had the same donor substrate specificity as EcWaaG, it was unable to complement an *E. coli* Δ*waaG* strain, indicating more remains to be understood about the function of PaWaaG *in vivo*. Overall, the structural and biochemical data presented here will be highly valuable for the future development of PaWaaG inhibitors for treating multidrug resistant *P. aeruginosa*.

## Experimental procedures

### Molecular cloning

The codon optimized sequence for *PaWaaG* and *PaWaaG-5mut* were purchased from GenScript and were cloned into the pET28a T7pCONS TIR2 plasmid (51) downstream of the coding sequence for the His-Thrombin tag, using the *in vivo* DNA assembly method (52). In brief, *PaWaaG* was PCR amplified using primers P1 and P2, while pET28a T7pCONS TIR2 (51) was PCR amplified using primers P3 and P4. The PCR products were *Dpn*I treated and agarose gel purified. Two microliters of both PCR purified insert and plasmid were mixed and allowed to incubate at room temperature for 10 min followed by transformation into *E. coli* MC1061. All PCRs were carried out with the Q5-polymerase (New England Biolabs). Oligonucleotide synthesis and DNA sequencing was performed by Eurofins Scientific (Eurofins genomics). All primers used in this study are listed in Table S1 and all coding sequences are listed in Table S2.

### Protein purification

His-tagged PaWaaG and PaWaaG-5mut were expressed in *E. coli* BL21 cells overnight at 18 °C using an LEX bioreactor. The cells were harvested and resuspended in lysis buffer (100 mM Tris–HCl pH 8.5, 500 mM NaCl, 10% glycerol, and 5 mM β-mercaptoethanol) and then lysed *via* sonication. The lysate was clarified by ultracentrifugation for 1 h at 40,000 rpm. PaWaaG and PaWaaG-5mut were purified to homogeneity using Immobilized metal affinity chromatography followed by size-exclusion chromatography using a Superdex 200 16/600 column. The proteins were exchanged into storage buffer containing 20 mM Tris–HCl pH 8.5, 300 mM NaCl, 10% glycerol and 0.5 mM tris(2-carboxyethyl)phosphine. Protein aliquots were then flash frozen in liquid nitrogen, and stored at −80 °C.

### PaWaaG crystallization

Aliquots of purified PaWaaG (11.5 mg/ml) were preincubated with 5 mM of either UMP, UDP-glucose, UDP-galactose, or UDP-GalNAc. The protein samples were crystallized *via* sitting drop vapor diffusion at 4 °C in the following conditions: 0.2 M lithium chloride, 0.1 M Mes pH 6.0, 20 % (v/v) PEG6000 (PaWaaG-UMP), condition A9 of Morpheus screen: 0.06 divalents, 0.1 M buffer system 3 pH 8.5, 30 % (v/v) P500MME_P20K (PaWaaG-UDP-glucose), 0.1 M PCTP pH 6.0, 25 % (v/v) PEG1500 (PaWaaG-UDP-galactose), 0.1 M MIB pH 4.0, 25 % (v/v) PEG1500 (PaWaaG-UDP-GalNAc). Protein crystals were soaked briefly in cryoprotectant solutions consisting of their respective growth condition supplemented with 5 mM of the relevant nucleotide and 20 % (v/v) glycerol, before flash freezing in liquid nitrogen.

### Data collection, structure determination, and refinement

X-ray diffraction data were collected on the BioMAX beamline at MAXIV (PaWaaG-UMP and PaWaaG-UDP-glucose datasets) and the I03 beamline of the Diamond Light Source (Oxford) (PaWaaG-UDP-glucose and PaWaaG-UDP-GalNAc datasets). All datasets were collected at 100 K using single crystals. Data indexing and integration were performed using DIALS (53), with the exception of the PaWaaG-UMP data which was processed using XDS (54). All data scaling was performed in AIMLESS (55) within the CCP4 suite (56). The structures were solved *via* molecular replacement with PHASER (57) using the monomer of *E. coli* WaaG (PDB ID: 2IV7) as the search model. Several rounds of manual model building and refinement were performed using Coot (58) and REFMAC5 (59) during which waters and ligands were incorporated into the structures. Data processing and refinement statistics are presented in Table 1. The coordinates and structure factors for PaWaaG-UMP, PaWaaG-UDP-glucose, PaWaaG-UDP-galactose, and PaWaaG-UDP-GalNAc were deposited in the PDB under the codes 8B5Q, 8B5S, 8B62, and 8B63, respectively.

### Sample preparation for solution NMR

Frozen aliquots of PaWaaG or PaWaaG-5mut in buffer containing glycerol were transferred into a glycerol free buffer by diluting and concentrating the samples three times using centrifugal concentrators (30 kDa MWCO) in buffer consisting of 20 mM Tris HCl pH 8.5, 300 mM NaCl and 500 μM tris(2-carboxyethyl)phosphine. After addition of 10 % D_2_O for field locking and 10 μM 2,2-dimethyl-2-silapentane-5-sulphonate for chemical shift referencing, the resulting concentrations of the NMR samples were 23 μM for PaWaaG and 20 μM for PaWaaG-5mut.

### NMR spectroscopy

1D ^1^H and ^31^P NMR spectra were recorded on a 600 MHz spectrometer using a room temperature BBI-probe with an outer phosphorus channel. The temperature was set to 298 K and calibrated using an external thermocouple. Proton spectra were recorded using 256 scans, a 2.3 s acquisition time and a 1 s recycle delay, giving measurement times of 14 min. The typical π/2 pulse length was 11 μs. Phosphorus spectra were recorded using 3000 or 12,000 scans (2 or 8 h measurement time), a 1.35 s acquisition time, 1 s recycle delay, and a 22.7 μs π/2 pulse length. Spectra were processed using TopSpin 3 (Bruker), applying 3 and 25 Hz line broadening for ^1^H and ^31^P, respectively, for display purposes. Proton spectra were referenced to 0 ppm for the methyl groups in 2,2-dimethyl-2-silapentane-5-sulphonate, and phosphorus spectra were indirectly referenced *via* the ^1^H frequency. Spectra were recorded on 600 μl samples of PaWaaG, before and after addition of 100 μM UDP-GalNAc, UDP-galactose, or UDP-glucose. For PaWaaG-5mut UDP-galactose was replaced by UDP-GlcNAc. Spectra were also recorded for the sugar donors in buffer without protein as controls to monitor for potential autohydrolysis.

### Complementation of phenotype in *E. coli* Δ*waaG* strain

The codon optimized coding sequences of His-TEV-EcWaaG and His-TEV-PaWaaG in pET22b and PaWaaG-5mut in pET24 were obtained from GenScript. The genes were subcloned into the pBAD/HisB expression plasmid using the *in vivo* DNA assembly method (52). In brief, His-TEV-*EcWaaG* was PCR amplified using primers P5 and P6, His-TEV-*PaWaaG* was PCR amplified using primers P5 and P7, pBAD/HisB-PAmCherry1 (plasmid #31931, obtained from Addgene) was PCR amplified using primers P8 and P9 and His-TEV-*PaWaaG-5mut* was PCR amplified using primers P10 and P11. The PCR products were *Dpn*I treated and 5 μl of insert and plasmid were mixed and allowed to incubate at room temperature for 10 min followed by transformation into *E. coli* MC1061. The transformation mix was plated onto LB-Agar supplemented with 100 μg/ml ampicillin, and incubated at 37 °C overnight. The resulting expression plasmids, pBAD-His-TEV-*EcWaaG* and pBAD-His-TEV-*PaWaaG* were sequenced (Eurofins genomics). The plasmids were then transformed into *E. coli* BW25113 (Δ*waaG*) and plated on LB agar containing 100 μg/ml ampicillin. Single colonies were then restreaked on LB agar containing 0.02 % (w/v) glucose and 100 μg/ml ampicillin, both with and without 0.0625 % (w/v) SDS. Plates were incubated at 37 °C overnight.

## Data availability

The protein structures presented in this paper have been deposited in the Protein Data Bank (PDB) under the accession codes 8B5Q, 8B5S, 8B62, and 8B63. All remaining data are contained within the article.

## Supporting information

This article contains supporting information.

## Conflict of interest

The authors declare that they have no conflicts of interest with the contents of this article.
