# Supplementary material for: Structural and functional insights into the Pseudomonas aeruginosa glycosyltransferase WaaG and the implications for lipopolysaccharide biosynthesis
Source: J Biol Chem. 2023 Sep 15;299(10):105256. doi: 10.1016/j.jbc.2023.105256 (PMC10579960; doi:10.1016/j.jbc.2023.105256)
Supplement: Supplemental Figs. S1–S6, and Tables S1 and S2 [file mmc1.docx]

**Structural and functional insights into *Pseudomonas aeruginosa* glycosyltransferase WaaG and the implications for lipopolysaccharide biosynthesis**

Emma R. Scaletti^1†^, Pontus Pettersson^1†^, Joan Patrick^1^, Patrick J. Shilling^1^, Robert Gustafsson Westergren^1^, Daniel O. Daley^1^, Lena Mäler^1^, Göran Widmalm^2^ and Pål Stenmark^1*^

^1^Department of Biochemistry and Biophysics, Stockholm University, SE-106 91 Stockholm, Sweden

^2^Department of Organic Chemistry, Arrhenius Laboratory, Stockholm University, SE-106 91 Stockholm, Sweden

^†^Authors contributed equally to this work

^*^Corresponding author. Correspondence and requests for materials should be addressed to Prof. Pål Stenmark, Department of Biochemistry and Biophysics, Stockholm University, SE‑106 91 Stockholm, Sweden, E-mail: [stenmark@dbb.su.se](mailto:stenmark@dbb.su.se)

*
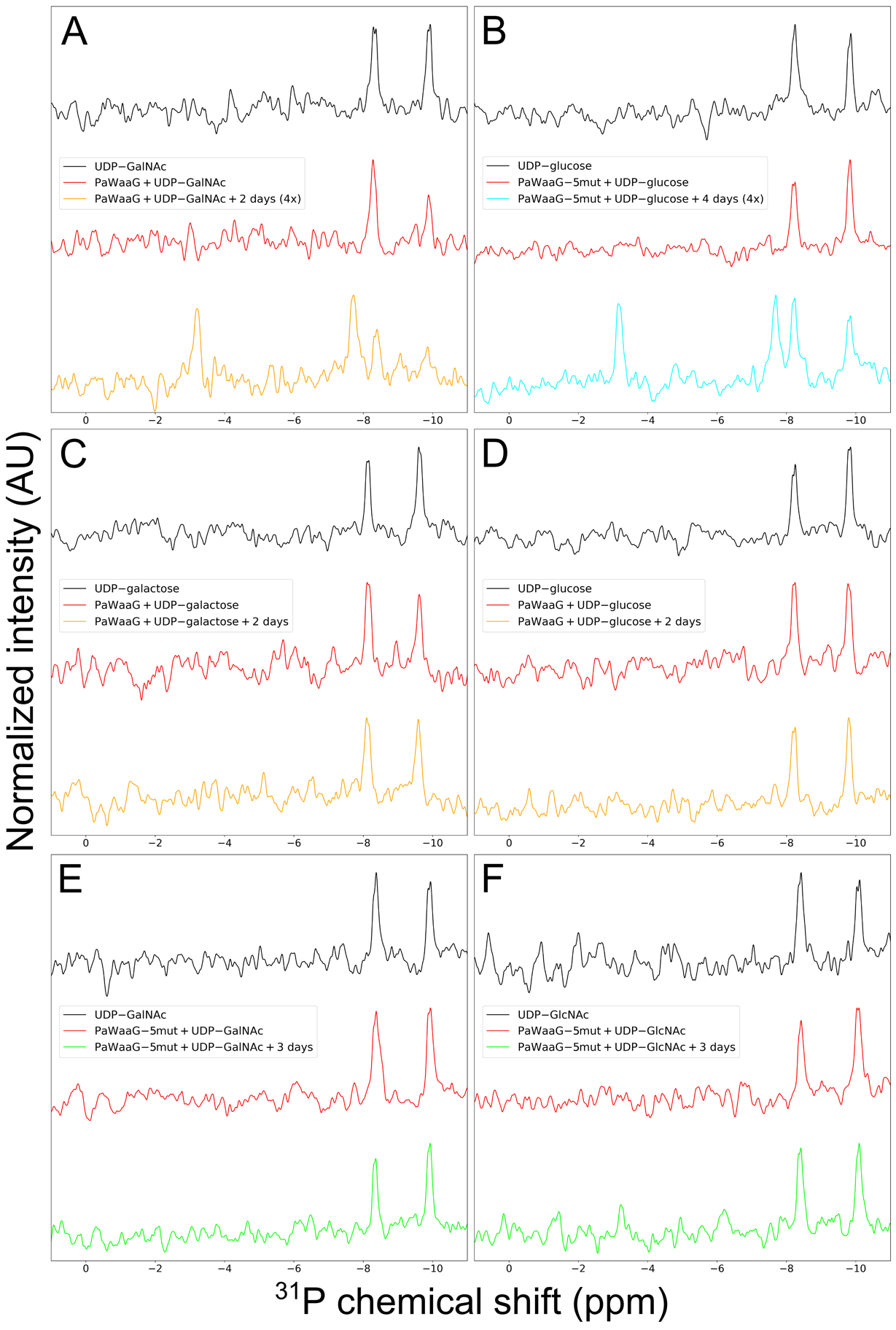
*

**Figure S1.** ^31^P NMR spectra of PaWaaG and PaWaaG-5mut with sugar donors. Panels A and B display the hydrolysis of UDP-GalNAc and UDP-glucose by PaWaaG and PaWaaG-5mut, respectively. Panels C-D and E-F show spectra of PaWaaG and PaWaaG-5mut, respectively, together with sugar donors that are not hydrolysed. All spectra have been normalized according to the highest peak in each spectrum and shifted vertically for display purposes. Spectra labelled “4×” were recorded using four times longer experimental time.

*
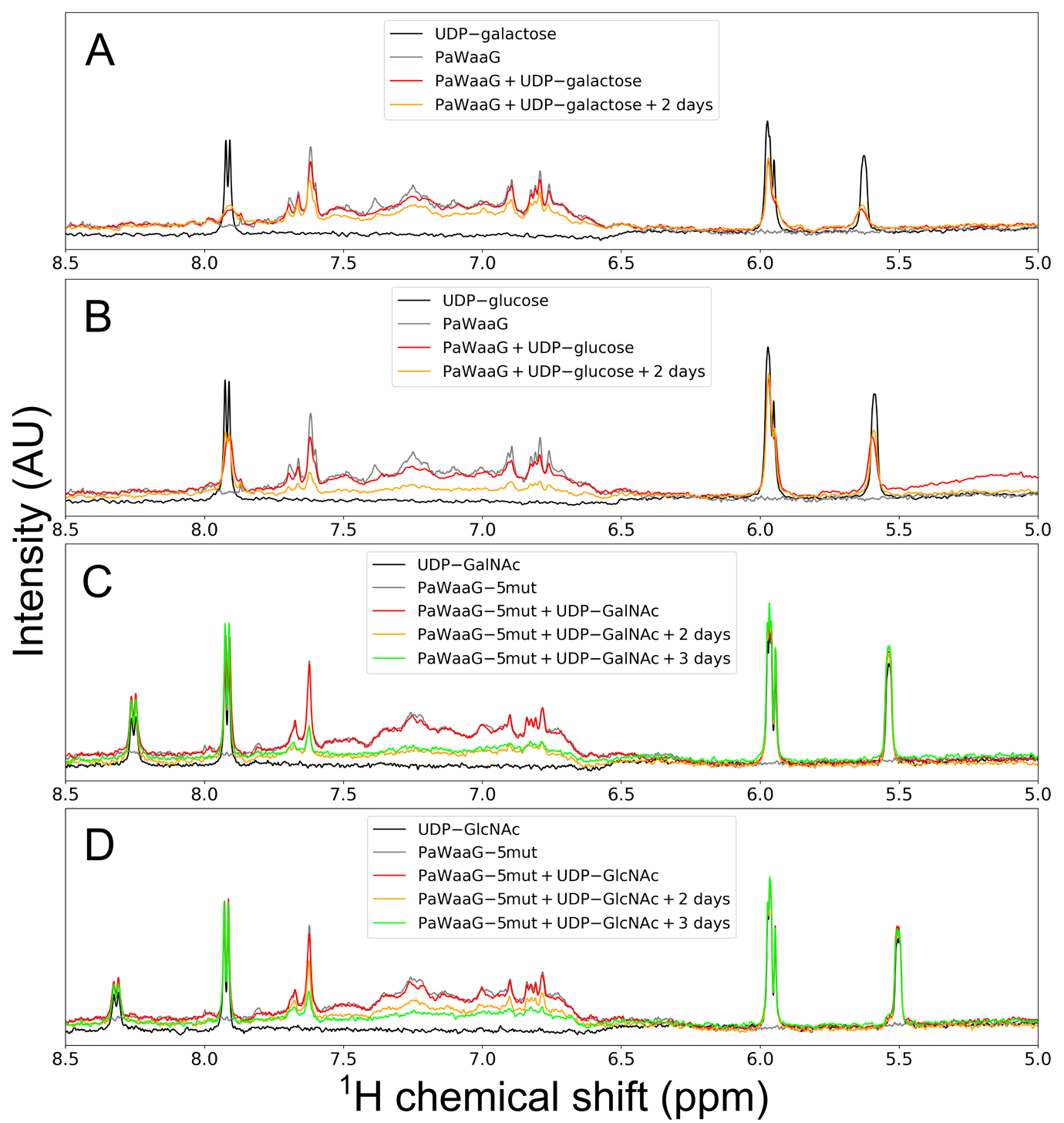
*

**Figure S2:** ^1^H NMR spectra of PaWaaG with (A) UDP-galactose or (B) UDP-glucose and PaWaaG-5mut with (C) UDP-GalNAc or (D) UDP-GlcNAc. Spectra of proteins together with sugar donors are colour coded according to the number of days after substrates were added. Spectra of sugar donors in buffer (black) and of proteins before addition of substrate (grey) are also shown.

**
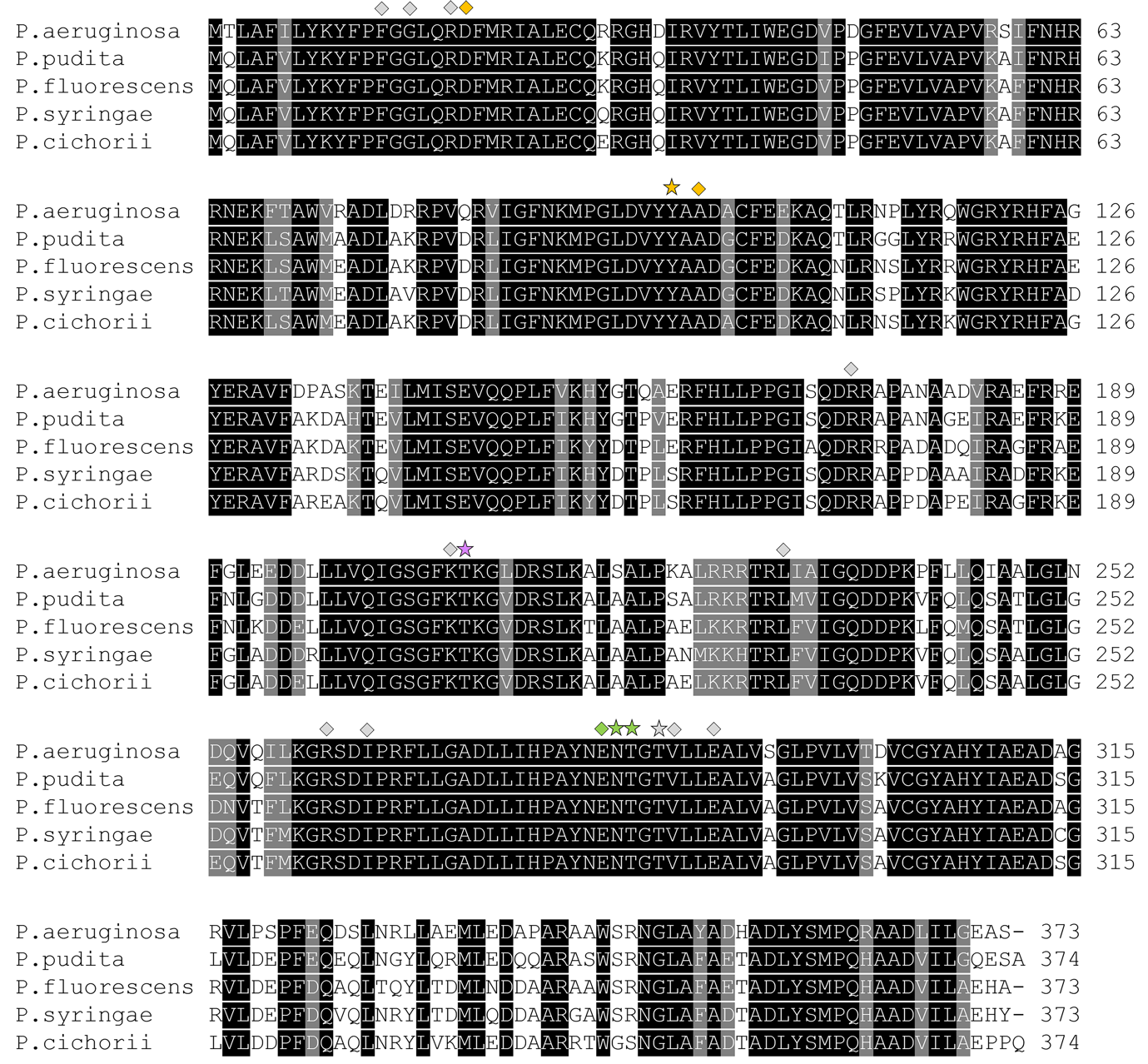
**

**Figure S3.** Sequence alignment of *P. aeruginosa* WaaG with other *Pseudomonas* species. The amino acids sequences of *P. aeruginosa* WaaG (UniProt: Q9HUF6), *P. pudita* WaaG (NCBI: WP_196179195.1), *P. fluorescens* WaaG (NCBI: WP_011332133.1), *P. syringae* WaaG (NCBI: WP_011105267.1) and *P. cichorii* WaaG (NCBI: WP_025258251.1) were aligned using Clustal Omega through the EBI webserver. The resulting alignment is coloured according to sequence similarity using BOXSHADE. Identical residues are shaded black, while dark grey shading indicates amino acids with conserved physicochemical properties. Residues important for the positioning of nucleotide sugars in PaWaaG are shown above the alignment. Light grey symbols indicate UMP/UDP interacting amino acids. Green symbols show residues that hydrogen bond with the glucose, galactose and GalNAc moieties of the nucleotide sugars. Yellow symbols indicate residues that hydrogen bond with galactose and GalNAc only. The purple symbol indicates a residue (Thr208, PaWaaG numbering) that is proposed to be important for the specificity of PaWaaG towards UDP-GalNAc. Stars indicate five amino acids that were mutated in the PaWaaG sequence to the equivalent EcWaaG residues to produce the PaWaaG-5mut mutant.


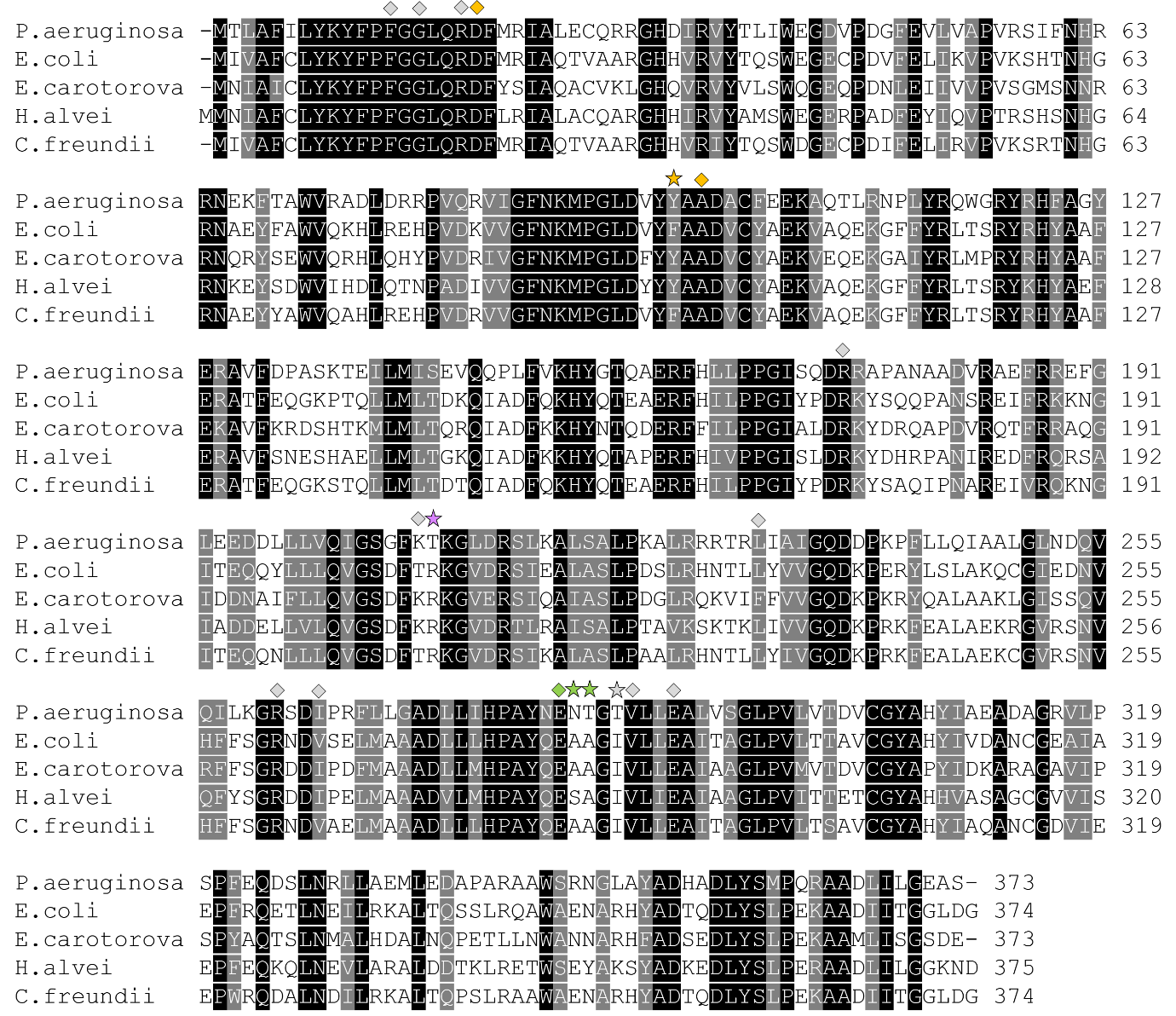


**Figure S4.** Sequence alignment of *P. aeruginosa* WaaG with homologues from other gram-negative bacteria. The amino acids sequences of *P. aeruginosa* WaaG (UniProt: Q9HUF6), *E. coli* WaaG (UniProt: B7L754), *E. carotorova* WaaG (NCBI: WP_110163099.1), *H. alvei* WaaG (NCBI: SCM52069.1) and *C. freundii* WaaG (NCBI: HAT2168144.1) were aligned using Clustal Omega through the EBI webserver. The resulting alignment is coloured according to sequence similarity using BOXSHADE. Identical residues are shaded black, while dark grey shading indicates amino acids with conserved physicochemical properties. Residues important for the positioning of nucleotide sugars in PaWaaG are shown above the alignment. Light grey symbols indicate UMP/UDP interacting amino acids. Green symbols show residues that hydrogen bond with the glucose, galactose and GalNAc moieties of the nucleotide sugars. Yellow symbols indicate residues that hydrogen bond with galactose and GalNAc only. The purple symbol indicates a residue (Thr208, PaWaaG numbering) that is proposed to be important for the specificity of PaWaaG towards UDP-GalNAc. Stars indicate five amino acids that were mutated in the PaWaaG sequence to the equivalent EcWaaG residues to produce the PaWaaG-5mut mutant.

**
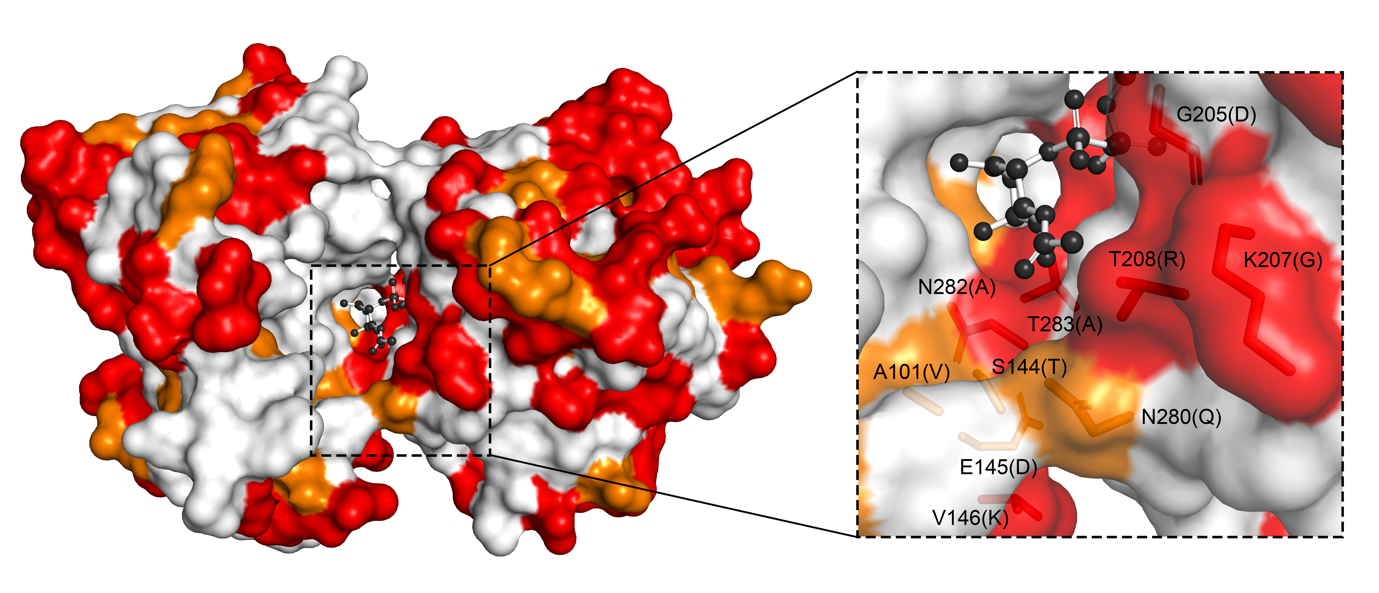
**

**Figure S5.** Structure of the PaWaaG-UDP-GalNAc monomer shown in surface representation (left), highlighting the amino acid differences between the enzyme and EcWaaG. UDP‑GalNAc is shown as a black ball‑and‑stick model. Red colouring indicates differences and orange colouring indicates differences with conserved physicochemical properties. A magnified view of the likely acceptor LPS binding site is also shown (right). Amino acid numbering refers to the PaWaaG structure. The amino acid indicated in brackets refers to the equivalent residue in the EcWaaG structure. Figures were produced with PyMOL (v.2.3.3, Schrödinger).

**
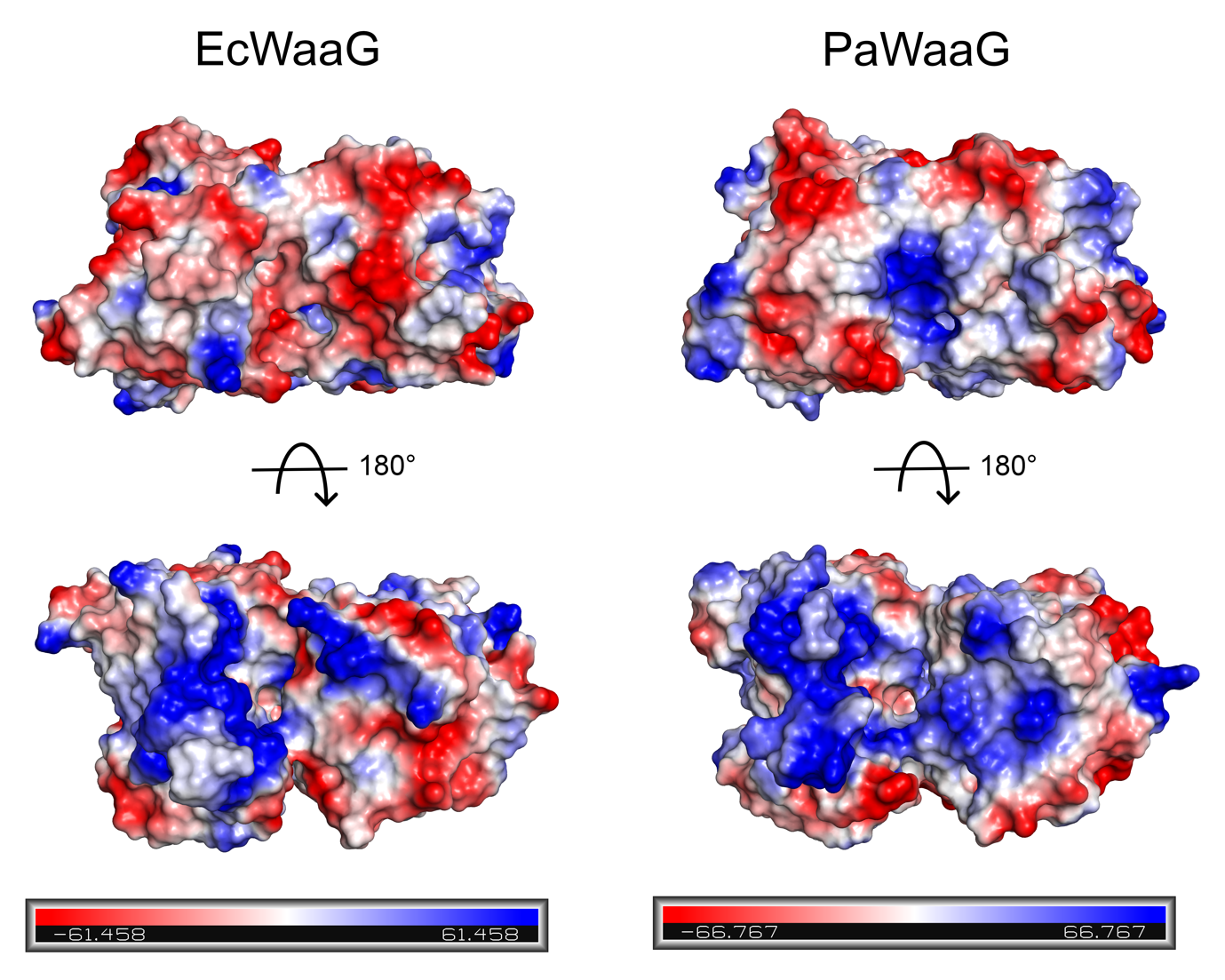
**

**Figure S6.** Molecular surface representations showing the electrostatic surface potentials of EcWaaG (PDB ID: 2iw1) and PaWaaG-UMP, positioned so that the corresponding areas of the surfaces are directly comparable. The positively and negatively charged surfaces are coloured blue and red, respectively, and the nonpolar/hydrophobic surfaces are coloured white. Figures were produced with PyMOL (v.2.3.3, Schrödinger).

**Table S1.** Oligonucleotides used for molecular cloning

| **Oligonucleotide identifier** | **Oligonucleotide name** | **Sequence (5’-3’)** |
| --- | --- | --- |
| P1 | WaaG_pET28a.Fwd | AGCGGCCTGGTGCCGCGCGGCAGCCATATGGCGACCCTGGCGTTTATTC |
| P2 | WaaG_pET28a.Rvs | ATCTCAGTGGTGGTGGTGGTGGTGCTCGAGTTAGCTCGCTTCGCCCAGA |
| P3 | pET28a_Thrombin.Rvs | CATATGGCTGCCGCGCGGCACCAGGC |
| P4 | p28_sfGFP_gibson.Fwd | CTCGAGCACCACCACCACCACCACTGAG |
| P5 | His.fwd | ATGCTGCACCACCACCACCACCAC |
| P6 | EcWaaG.rev | CAGCCAAGCTTCGAATTCTTAACCATCCAGGCCACCGG |
| P7 | PaWaaG.rev | CAGCCAAGCTTCGAATTCTTAGCTCGCTTCGCCCAG |
| P8 | pBAD-HisB.Fwd | GAATTCGAAGCTTGGCTG |
| P9 | His.rev | GTGGTGGTGGTGGTGCAGCATGGTTAATTCCTCCTGTTAGCCC |
| P10 | TEV_3mut.fwd | CCTGTACTTCCAGGGTGCGACCCTGGCGTTCATCCTGTAC |
| P11 | PaWaaG_mut.rev | CAGCCAAGCTTCGAATTCTTAGCTCGCCTCGCCCAG |

**Table S2.** Sequences expressed in the study

| **Construct** | **Sequence** |
| --- | --- |
| His-Thrombin-PaWaaG | ATGCAGCTTAGCCATCATCATCATCATCACAGCAGCGGCCTGGTGCCGCGCGGCAGCCATATGGCGACCCTGGCGTTTATTCTGTACAAGTATTTCCCGTTTGGTGGCCTGCAGCGTGACTTCATGCGTATCGCGCTGGAATGCCAACGTCGTGGCCACGATATCCGTGTGTATACCCTGATTTGGGAGGGTGACGTTCCGGATGGCTTTGAAGTGCTGGTTGCGCCGGTGCGTAGCATCTTCAACCACCGTCGTAACGAGAAATTTACCGCGTGGGTTCGTGCGGACCTGGATCGTCGTCCGGTGCAACGTGTTATTGGTTTCAACAAGATGCCGGGCCTGGACGTGTACTATGCGGCGGATGCGTGCTTTGAGGAAAAAGCGCAGACCCTGCGTAACCCGCTGTACCGTCAATGGGGTCGTTATCGTCACTTCGCGGGCTACGAGCGTGCGGTTTTTGACCCGGCGAGCAAGACCGAGATCCTGATGATTAGCGAAGTGCAGCAACCGCTGTTCGTTAAACACTACGGTACCCAGGCGGAACGTTTTCACCTGCTGCCGCCGGGTATCAGCCAAGACCGTCGTGCGCCGGCGAACGCGGCGGATGTGCGTGCGGAGTTCCGTCGTGAATTTGGTCTGGAGGAAGACGATCTGCTGCTGGTTCAGATTGGTAGCGGCTTCAAGACCAAAGGCCTGGATCGTAGCCTGAAAGCGCTGAGCGCGCTGCCGAAAGCGCTGCGTCGTCGTACCCGTCTGATCGCGATTGGTCAGGACGATCCGAAGCCGTTCCTGCTGCAAATCGCGGCGCTGGGTCTGAACGATCAGGTGCAAATCCTGAAAGGCCGTAGCGACATTCCGCGTTTTCTGCTGGGTGCGGATCTGCTGATTCACCCGGCGTACAACGAGAACACCGGCACCGTGCTGCTGGAAGCGCTGGTTAGCGGTCTGCCGGTGCTGGTTACCGACGTGTGCGGCTACGCGCACTATATTGCGGAAGCGGATGCGGGTCGTGTTCTGCCGAGCCCGTTCGAACAGGACAGCCTGAACCGTCTGCTGGCGGAGATGCTGGAGGATGCGCCGGCGCGTGCGGCGTGGAGCCGTAACGGTCTGGCGTATGCGGACCACGCGGATCTGTACAGCATGCCGCAACGTGCGGCGGACCTGATTCTGGGCGAAGCGAGCTAA |
| His-TEV-PaWaaG | ATGCTGCACCACCACCACCACCACCCGGCGAGCGACTATGATATCCCGACCACCGAGAACCTGTACTTCCAGGGTGCGACCCTGGCGTTTATTCTGTACAAGTATTTCCCGTTTGGTGGCCTGCAGCGTGACTTCATGCGTATCGCGCTGGAATGCCAACGTCGTGGCCACGATATCCGTGTGTATACCCTGATTTGGGAGGGTGACGTTCCGGATGGCTTTGAAGTGCTGGTTGCGCCGGTGCGTAGCATCTTCAACCACCGTCGTAACGAGAAATTTACCGCGTGGGTTCGTGCGGACCTGGATCGTCGTCCGGTGCAACGTGTTATTGGTTTCAACAAGATGCCGGGCCTGGACGTGTACTATGCGGCGGATGCGTGCTTTGAGGAAAAAGCGCAGACCCTGCGTAACCCGCTGTACCGTCAATGGGGTCGTTATCGTCACTTCGCGGGCTACGAGCGTGCGGTTTTTGACCCGGCGAGCAAGACCGAGATCCTGATGATTAGCGAAGTGCAGCAACCGCTGTTCGTTAAACACTACGGTACCCAGGCGGAACGTTTTCACCTGCTGCCGCCGGGTATCAGCCAAGACCGTCGTGCGCCGGCGAACGCGGCGGATGTGCGTGCGGAGTTCCGTCGTGAATTTGGTCTGGAGGAAGACGATCTGCTGCTGGTTCAGATTGGTAGCGGCTTCAAGACCAAAGGCCTGGATCGTAGCCTGAAAGCGCTGAGCGCGCTGCCGAAAGCGCTGCGTCGTCGTACCCGTCTGATCGCGATTGGTCAGGACGATCCGAAGCCGTTCCTGCTGCAAATCGCGGCGCTGGGTCTGAACGATCAGGTGCAAATCCTGAAAGGCCGTAGCGACATTCCGCGTTTTCTGCTGGGTGCGGATCTGCTGATTCACCCGGCGTACAACGAGAACACCGGCACCGTGCTGCTGGAAGCGCTGGTTAGCGGTCTGCCGGTGCTGGTTACCGACGTGTGCGGCTACGCGCACTATATTGCGGAAGCGGATGCGGGTCGTGTTCTGCCGAGCCCGTTCGAACAGGACAGCCTGAACCGTCTGCTGGCGGAGATGCTGGAGGATGCGCCGGCGCGTGCGGCGTGGAGCCGTAACGGTCTGGCGTATGCGGACCACGCGGATCTGTACAGCATGCCGCAACGTGCGGCGGACCTGATTCTGGGCGAAGCGAGCTAA |
| His-TEV-EcWaaG | ATGCTGCACCACCACCACCACCACCCGGCGAGCGACTACGATATCCCGACCACCGAGAACCTGTACTTCCAGGGTGCGATTGTTGCGTTTTGCCTGTACAAGTATTTCCCGTTTGGTGGCCTGCAACGTGACTTTATGCGTATTGCGAGCACCGTGGCGGCGCGTGGTCACCACGTGCGTGTTTACACCCAGAGCTGGGAGGGTGATTGCCCGAAGGCGTTTGAACTGATTCAAGTGCCGGTTAAAAGCCACACCAACCACGGTCGTAACGCGGAGTACTATGCGTGGGTTCAGAACCACCTGAAGGAACACCCGGCGGACCGTGTGGTTGGTTTCAACAAAATGCCGGGCCTGGACGTGTACTTTGCGGCGGATGTTTGCTATGCGGAGAAGGTGGCGCAAGAAAAAGGCTTCCTGTATCGTCTGACCAGCCGTTACCGTCACTATGCGGCGTTCGAGCGTGCGACCTTTGAACAGGGCAAGAGCACCAAACTGATGATGCTGACCGACAAGCAAATCGCGGATTTCCAGAAACACTACCAAACCGAGCCGGAACGTTTTCAGATCCTGCCGCCGGGCATTTACCCGGACCGTAAGTATAGCGAGCAGATCCCGAACAGCCGTGAAATTTATCGTCAAAAGAACGGCATCAAAGAACAGCAAAACCTGCTGCTGCAGGTTGGTAGCGACTTTGGTCGTAAAGGCGTGGATCGTAGCATTGAAGCGCTGGCGAGCCTGCCGGAAAGCCTGCGTCACAACACCCTGCTGTTCGTGGTTGGTCAAGATAAGCCGCGTAAATTTGAGGCGCTGGCGGAAAAACTGGGTGTGCGTAGCAACGTTCACTTCTTTAGCGGCCGTAACGACGTTAGCGAGCTGATGGCGGCGGCGGATCTGCTGCTGCACCCGGCGTACCAGGAAGCGGCGGGTATCGTGCTGCTGGAAGCGATCACCGCGGGTCTGCCGGTGCTGACCACCGCGGTTTGCGGTTACGCGCACTATATCGCGGATGCGAACTGCGGCACCGTTATTGCGGAACCGTTCAGCCAGGAGCAACTGAACGAAGTGCTGCGTAAAGCGCTGACCCAAAGCCCGCTGCGTATGGCGTGGGCGGAGAACGCGCGTCACTACGCGGACACCCAAGATCTGTATAGCCTGCCGGAAAAAGCGGCGGACATCATTACCGGTGGCCTGGATGGTTAA |
| His-TEV-PaWaaG_5mut | ATGCTGCACCACCACCACCACCACCCGGCGAGCGACTACGATATCCCGACCACCGAGAACCTGTATTTCCAGGGCGCGACCCTGGCGTTCATCCTGTACAAGTATTTCCCGTTTGGTGGCCTGCAGCGCGACTTTATGCGTATTGCGCTGGAGTGCCAACGTCGCGGCCACGATATCCGTGTGTATACCCTGATTTGGGAGGGTGACGTTCCGGATGGCTTCGAAGTGCTGGTTGCGCCGGTGCGTAGCATCTTCAACCATCGTCGCAATGAAAAATTTACCGCGTGGGTTCGTGCGGACCTGGATCGTCGCCCGGTGCAGCGTGTTATTGGTTTCAACAAGATGCCGGGCCTGGACGTGTACttcGCGGCGGATGCGTGCTTTGAGGAAAAAGCGCAGACCCTGCGCAATCCGCTGTACCGTCAATGGGGTCGCTATCGTCACTTCGCGGGCTACGAGCGTGCGGTTTTTGACCCGGCGAGCAAGACCGAGATCCTGATGATTAGCGAAGTGCAGCAACCGCTGTTCGTTAAACACTACGGTACCCAGGCGGAACGTTTTCATCTGCTGCCGCCGGGTATCAGCCAGGACCGTCGCGCGCCGGCGAACGCGGCGGATGTGCGTGCGGAGTTCCGTCGCGAATTTGGTCTGGAGGAAGACGATCTGCTGCTGGTTCAGATTGGTAGCGGCTTCAAGcgtAAAGGCCTGGATCGTAGCCTGAAAGCGCTGAGCGCGCTGCCGAAAGCGCTGCGTCGCCGTACCCGTCTGATTGCGATTGGTCAGGACGATCCGAAGCCGTTCCTGCTGCAAATCGCGGCGCTGGGTCTGAACGATCAGGTGCAAATCCTGAAAGGCCGCAGCGACATTCCGCGTTTTCTGCTGGGTGCGGATCTGCTGATTCACCCGGCGTATAACGAGgcggcgGGCattGTGCTGCTGGAAGCGCTGGTTAGCGGTCTGCCGGTGCTGGTTACCGACGTGTGCGGCTACGCGCATTATATTGCGGAGGCGGATGCGGGTCGTGTTCTGCCGAGCCCGTTTGAACAGGACAGCCTGAACCGTCTGCTGGCGGAGATGCTGGAAGATGCGCCGGCGCGTGCGGCGTGGAGCCGTAATGGTCTGGCGTATGCGGACCACGCGGATCTGTACAGCATGCCGCAACGTGCGGCGGACCTGATCCTGGGCGAGGCGAGCTAA |

*His-TEV- sequence underlined
